# Multi-immersion open-top light-sheet microscope for high-throughput imaging of cleared tissues

**DOI:** 10.1038/s41467-019-10534-0

**Published:** 2019-07-04

**Authors:** Adam K. Glaser, Nicholas P. Reder, Ye Chen, Chengbo Yin, Linpeng Wei, Soyoung Kang, Lindsey A. Barner, Weisi Xie, Erin F. McCarty, Chenyi Mao, Aaron R. Halpern, Caleb R. Stoltzfus, Jonathan S. Daniels, Michael Y. Gerner, Philip R. Nicovich, Joshua C. Vaughan, Lawrence D. True, Jonathan T. C. Liu

**Affiliations:** 1 Department of Mechanical Engineering, University of Washington, Seattle, WA 98195 USA; 2 Department of Pathology, University of Washington, Seattle, WA 98195 USA; 3 Department of Chemistry, University of Washington, Seattle, WA 98195 USA; 4 Department of Immunology, University of Washington, Seattle, WA 98109 USA; 5 Applied Scientific Instrumentation, Eugene, OR 97402 USA; 6 Allen Institute for Brain Science, Seattle, WA 98109 USA; 7 Department of Physiology and Biophysics, University of Washington, Seattle, WA 98195 USA

**Keywords:** Optical imaging, Cancer, Neuroscience, Systems biology, Light-sheet microscopy

## Abstract

Recent advances in optical clearing and light-sheet microscopy have provided unprecedented access to structural and molecular information from intact tissues. However, current light-sheet microscopes have imposed constraints on the size, shape, number of specimens, and compatibility with various clearing protocols. Here we present a multi-immersion open-top light-sheet microscope that enables simple mounting of multiple specimens processed with a variety of clearing protocols, which will facilitate wide adoption by preclinical researchers and clinical laboratories. In particular, the open-top geometry provides unsurpassed versatility to interface with a wide range of accessory technologies in the future.

## Abstract (web summary)

Light-sheet microscopes are increasingly used for imaging cleared tissues, but have imposed constraints on sample geometries and protocols. Here the authors present a multi-immersion open-top light-sheet microscope to overcome these limitations and enable high-throughput imaging of samples processed with various clearing protocols.

## Introduction

Recent advances in tissue clearing have provided unprecedented visual access to structural features and molecular targets within large intact specimens^1^. These clearing approaches have the potential to accelerate new discoveries across diverse fields of research, such as neuroscience and developmental biology, as well as the potential to develop new clinical assays based on three-dimensional (3D) anatomic pathology^2–5^ (Supplementary Fig. 1). However, fully harnessing the benefits of tissue clearing requires user-friendly and versatile microscopes for 3D imaging of a variety of content-rich specimens.

Over the past decade, light-sheet fluorescence microscopy (LSFM) has emerged as the technique of choice for fast and gentle 3D microscopy of relatively transparent specimens^6,7^. In LSFM, a thin sheet of light illuminates a specimen such that fluorescence is selectively excited within a single optical section. The fluorescence is imaged onto a high-speed camera in the direction perpendicular to the light sheet. The initial LSFM architecture, known as selective-plane illumination microscopy (SPIM), was purposefully designed for imaging small model organisms, often living, over long timescales^8^. However, in recent years, LSFM has also been harnessed for 3D microscopy of larger cleared tissues, where high imaging speed and lower rates of photobleaching are the main advantages over alternative microscopy methods^9–11^. Many early LSFM systems that were designed to image cleared tissues, such as ultramicroscopy (UM) and CLARITY-optimized light-sheet microscopy (COLM), imaged specimens from above, with either air objectives or immersion objectives oriented downward toward (or into) a large liquid-filled chamber^9,10^. While this geometry is suitable for imaging moderately sized specimens such as mouse brains with dual-sided illumination for increased light penetration, the specimens are laterally constrained by the chamber and the arrangement of the objectives.

More recent LSFM architectures that allow for laterally unconstrained imaging include dual-inverted selective-plane illumination microscopy (diSPIM) and light-sheet theta microscopy (LSTM)^11,12^. With diSPIM, the illumination and collection objectives are oriented at 45 ° with respect to the vertical axis and dipped downward into a liquid reservoir containing a cleared specimen. With LSTM, a high-magnification collection objective is dipped downward in the vertical direction into a liquid reservoir, with a pair of longer working distance objectives (oriented at 60 ° with respect to the vertical axis) to generate the light sheet.

While both diSPIM and LSTM enable laterally unconstrained imaging, there are several drawbacks:^1^ When fully immersed, cleared tissues equilibrate to a similar density as the surrounding medium, which often causes the tissues to float and drift (Supplementary Fig. 2)^2^. Specimens sit within a large reservoir of immersion media, which results in inter-specimen contamination and dilution of reagents^3^. Immersion objectives are often incompatible with the corrosive organic solvents (e.g., dibenzyl ether, benzyl alcohol, and benzyl benzoate) used in certain tissue-clearing protocols^4^. Specimens are imaged from above, which makes it difficult to rely upon gravity or gentle pressure to flatten the top surfaces of the specimens (if so desired).

In order to improve the ease-of-use and throughput of LSFM, open-top light-sheet (OTLS) microscopes have been proposed, where laterally unconstrained imaging is enabled by orienting the illumination and collection objectives at 45 ° with respect to the vertical axis and placing them below (rather than above) a cleared specimen^5,13–15^. The open-top geometry mimics a flatbed document scanner, enabling multiple specimens to be simply placed atop a transparent specimen holder without having to be fully immersed, such that gravity and surface tension work to immobilize the specimen, and where gentle pressure can also be applied from above to flatten the specimen (if so desired). Not only does this configuration mitigate the challenges listed in the previous paragraph, but the OTLS system also allows for a variety of potential accessory devices to be positioned on the specimen stage or above the specimen(s). One downside of the OTLS configuration, similar to the diSPIM system, is that orienting the objectives at an oblique angle reduces their usable working distance, which in turn limits the imaging depth of the OTLS system (see Supplementary Fig. 3). This is in contrast to LSTM, where the working distance of the collection objective is fully utilized. A summary of the advantages and disadvantages of these LSFM architectures is provided in Supplementary Fig. 4.

While the OTLS architecture is highly versatile and easy to use, it also presents unique optical challenges since achieving laterally unconstrained imaging requires off-axis illumination and collection beams that must be index-matched into and out of the holder and specimen to prevent aberrations. To enable aberration-free imaging, both a water prism and solid-immersion lens (SIL) have previously been proposed to serve as an interface for the illumination and collection beams as they transition from air into a higher-index medium: the former by providing a normal interface to mitigate off-axis aberrations, and the latter by providing a wavefront-matched interface to prevent both off-axis and spherical aberrations^5,13,14^. While these solutions address the issue of off-axis aberrations, they limit specimen scanning to 2D rather than a desired 3D (i.e., the specimens cannot be physically lowered without colliding with the water prism or SIL). In addition, these techniques are not readily compatible with a range of refractive indices, as the specimen must precisely match the refractive index of the water prism or SIL material.

Here, we develop an easy-to-use multi-immersion open-top light-sheet (OTLS) microscope that overcomes the limitations of previous LSFM systems, enabling high-throughput 3D imaging of one or more specimens prepared with any published clearing protocol^5,13,15^. Our system simplifies specimen mounting and imposes minimal constraints on specimen size/shape, which will facilitate wider adoption by preclinical and clinical researchers. In particular, we demonstrate sub-micron resolution at an imaging speed of ~1 mm^3^/min, with a maximum usable imaging depth of 0.5 cm, and over a lateral area of up to 10 × 10 cm.

## Results

### Multi-immersion open-top light-sheet microscope

**Fig. 1:**
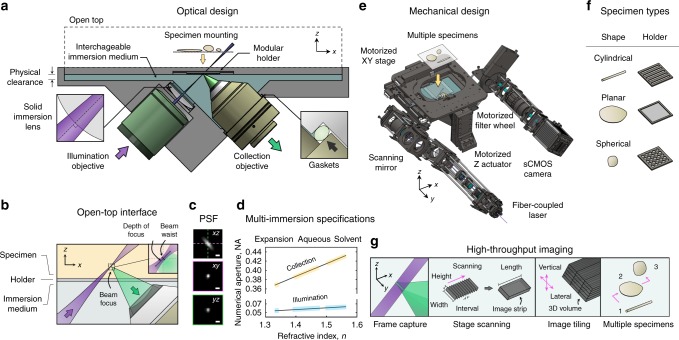
Multi-immersion open-top light-sheet (OTLS) microscope. **a** The system enables simple mounting of multiple specimens with modular transparent holders. Illumination and collection objectives are located underneath the specimen holders and are separated by a liquid reservoir filled with an interchangeable immersion medium. **b** The off-axis illumination light sheet and collected fluorescence travel obliquely through the immersion media, holder, and specimen. Aberrations are minimized by precisely matching the refractive index of all three materials, and by utilizing the wavefont-matching properties of a solid-immersion lens (SIL) along the illumination path. The depth-of-focus and beam waist of the light sheet are depicted in the inset (upper right). The point spread function (PSF) of the system (scale bars: 1 μm) and refractive-index-dependent numerical aperture (NA) of the illumination and collection beams are shown in (**c**) and (**d**). **e** The mechanical design of the system includes a motorized XY stage, motorized Z actuators, motorized filter wheel, scanning mirror, computer-controlled multi-wavelength fiber-coupled laser package, and sCMOS camera, all of which enable high-throughput automated imaging of multiple specimens simply placed on a flat plate, or placed within a diverse assortment of transparent holder designs (**f**). **g** Volumetric imaging is achieved by using a combination of stage scanning and lateral/vertical tiling

An air objective delivers an illumination light sheet through an immersion chamber, holder, and into a specimen, where fluorescence is generated and then collected by a multi-immersion objective in the direction orthogonal to the light sheet (Fig. 1a; Supplementary Movie 1). The multi-immersion capability of our OTLS system spans the refractive index range of all published tissue clearing methods (including expansion, aqueous, and solvent-based protocols). A key to achieving this is by precisely matching the refractive index of the immersion medium, holder material, and specimen (Fig. 1b). A plano-convex lens with a center of curvature that is coincident with the focus of the illumination beam acts as a solid-immersion lens (SIL), preventing spherical and off-axis aberrations from occurring along the illumination beam and obviating the need for optical realignment when the immersion medium is changed. The point spread function (PSF) of the system is shown in Fig. 1c, in which sub-micron resolution is achieved lateral to the collection axis. Resolution along the collection axis (~3.5 μm) is comparable with the thickness of a typical slide-mounted histology section. Note that the numerical apertures (NA) of both the illumination and collection beams are proportional to the matched refractive index of the immersion medium, holder material, and specimen (Fig. 1d). The open-top architecture (Fig. 1e) enables fast and simple mounting of multiple specimens with diverse shapes, such as human biopsies, thin tissue slices, and whole mouse organs in modular holders, where light pressure can be applied from above without interfering with the optical components below (Fig. 1f). Large-volume imaging at a speed of ~1 mm^3^ min^−1^ is achieved entirely through stage scanning, in which a series of adjacent volumetric image strips (scanned in the *x* dimension) are tiled in the lateral (*y*) and vertical (*z*) directions (Fig. 1g; Supplementary Movie 2). The working distance of the collection objective allows for an imaging depth of up to 0.5 cm. In comparison with our previously published OTLS prototype^5^, our new OTLS system exhibits:^1^ improved axial and lateral resolution (an order-of-magnitude reduction in the focal volume)^2^, ~20 × greater imaging depth^3^, mitigation of shadowing artifacts, and^4^ multi-immersion capabilities (Supplementary Fig. 5).

### Holder design for OTLS imaging

**Fig. 2:**
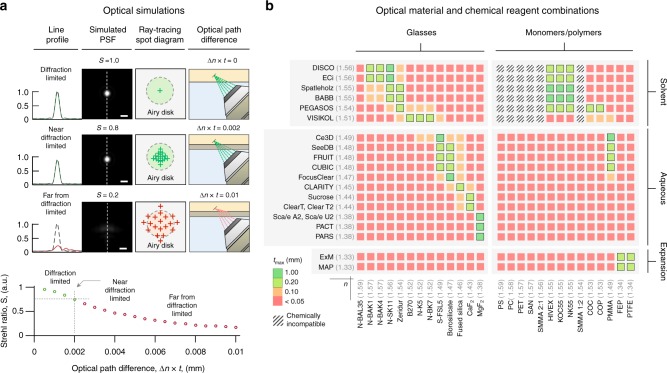
Holder design for OTLS imaging. **a** Optical simulations of the system’s point spread function (PSF) and Strehl ratio (*S*), and ray-tracing spot diagrams, are shown for scenarios in which the optical path difference (Δ*n* × *t*) is negligible, small, or large, which results in diffraction-limited (*S* ~ 1.0), near-diffraction-limited (*S* > 0.8), or aberrated (*S* < 0.8) imaging performance, respectively (scale bars: 1 μm). The dependence of the Strehl Ratio, *S*, as a function of Δ*n* × *t* is plotted, indicating that for diffraction-limited imaging, the condition that Δ*n* × *t* < 0.002 mm should be maintained. Based on this condition, potential glass and monomer/polymer holder materials are shown in (**b**). The color scale indicates the maximum material thickness, *t*_max_, that is allowed based upon the intrinsic mismatch, Δ*n*, of those materials with published clearing protocols. Chemically incompatible combinations of materials and chemical reagents are also indicated

Since precise index matching of the immersion media, holder, and specimen are necessary for aberration-free imaging, we performed optical simulations (Fig. 2a) to explore the tolerance of the system to the optical path difference (Δ*n* × *t*) introduced by a holder with a refractive index mismatch, Δ*n*, and thickness, *t*. We quantified the Strehl Ratio, *S*, of the system as a function of Δ*n* × *t*, and determined that Δ*n* × *t* < 0.002 mm is necessary to achieve near-diffraction-limited imaging (*S* > 0.8). Note that this condition is a function of NA, as shown in Supplementary Fig. 6. Based on these findings, we surveyed glasses and monomers/polymers as potential holder materials, and determined the maximum-allowed thickness, *t*_max_, based upon the intrinsic mismatch, Δ*n*, between these materials and published clearing protocols (Fig. 2b). These chemical–reagent and material combinations enable multi-immersion imaging with our OTLS system, and are beneficial for other optical systems that require aberration-free transmission of light through tilted or irregular interfaces. To validate the multi-immersion capabilities of our system, we fabricated customized holders for a variety of clearing protocols spanning solvent, aqueous, and expansion-based protocols, and performed high-throughput imaging of diverse tissue types and shapes (a summary of the imaging parameters and immersion media for all specimens are shown in Supplementary Tables 1, 2).

### Multi-immersion and multi-specimen imaging

**Fig. 3:**
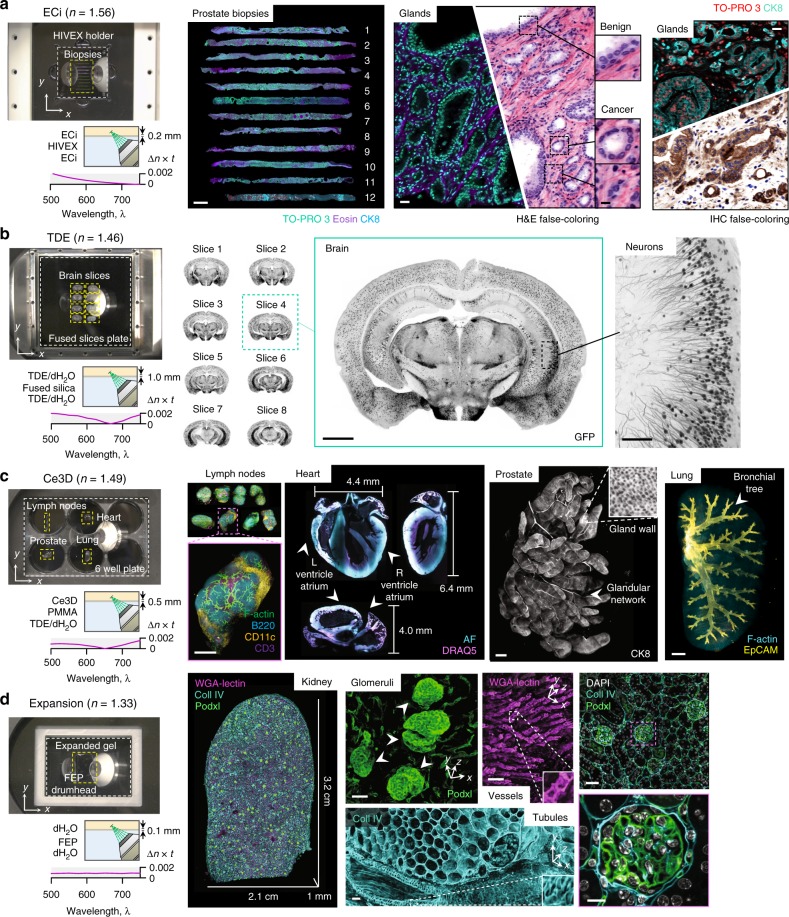
Multi-immersion and multi-specimen imaging. **a** OTLS imaging of 12 ECi-cleared human prostate biopsies (scale bar: 1 mm) placed within a multi-biopsy holder. Zoomed-in views illustrate the complex 3D structure of benign and malignant glands (scale bars: 25μm and 10 μm). Images can be false-colored to mimic the appearance of conventional chromogen-based (absorption-based) H&E and IHC histopathology. **b** High-throughput imaging of eight TDE-cleared mouse brain slices placed on a 10 × 10 -cm glass plate (scale bar: 1 mm). A higher-magnification region of interest demonstrates the ability to visualize individual neurons (scale bar: 100 μm). **c** Whole-organ imaging of Ce3D-cleared mouse lymph nodes, heart, prostate, and lung placed within an multi-well plate (scale bars: 1 mm). **d** Large-scale imaging of an expanded thick-kidney slice placed on a teflon drumhead. High-resolution regions of interest show individual glomeruli (scale bar: 40 μm), vessels (scale bar: 80 μm), and tubules (scale bar: 50 μm). A multi-channel zoom-in with DAPI counterstaining demonstrates the increased resolution due to expansion (scale bars: 100μm and 20 μm). The scale bars for the entire expanded kidney indicates the physical size of the expanded specimen, whereas the scale bars for the insets indicate the dimensions of the native unexpanded tissue

Solvent-based protocols involve dehydration of tissue specimens and replace the water with organic reagents of a higher refractive index (*n* = 1.51–1.56). These solvents are optically compatible with several high-index glasses. Unfortunately, these higher-index glasses also have high Abbe numbers (i.e., a low variation in refractive index versus wavelength) compared with organic solvents, which typically have low Abbe numbers. In addition, glasses, which are brittle, must be relatively thick and are not easily machined. Therefore, we explored the use of several monomers/polymers. Despite being optically compatible, we observed that styrene-based polymers (e.g., PS, SMMA, and SAN) were all destroyed after exposure to solvent-based clearing reagents. However, we identified three optically and chemically compatible resin-based monomers (HIVEX, NK55, and KOC55, used for manufacturing eyeglass lenses) that are ideal for many solvent-based clearing protocols, including DISCO, BABB, and ECi^16–20^. We demonstrated the potential clinical utility of our system by imaging multiple human prostate biopsies in toto (Fig. 3a; Supplementary Movies 3, 4). We found that ECi-clearing (*n* = 1.56) is well suited for this application due to its clearing efficacy and low toxicity^21^. A custom HIVEX holder (*n* = 1.55) was machined with channels to accommodate multiple biopsies (13 in this case) and to keep them aligned and parallel. Due to the effective tissue clearing of ECi and precise refractive index matching with the HIVEX holder, we were able to resolve sub-nuclear features in benign and malignant prostate glands throughout the entire 1-mm diameter of all biopsies. For example, epithelial cells within benign glands have inconspicuous nucleoli, whereas carcinoma cells within malignant glands exhibit enlarged nuclei with prominent nucleoli. As noted by us and others, this ability to visualize the 3D structure of human cancers should improve prognostication and treatment decisions^3,5,22^.

Aqueous-based protocols typically involve removing lipids using detergents, followed by immersion in diluted water-soluble reagents, resulting in a refractive index range of *n* = 1.38–1.49^23^. For most aqueous-based protocols, there is an optically compatible glass, such that Δ*n* ≤ 0.01. For example, fused silica (*n* = 1.46) is well-suited for several protocols, including CLARITY^2,24^. However, most transparent monomers/polymers (with the exception of PMMA) have either a high (*n* > 1.49) or low (*n* < 1.38) refractive index and are therefore not optically compatible with aqueous-based protocols. To demonstrate compatibility of our system with aqueous protocols, we cleared adjacent slices of a mouse brain (200-μm thick) using simple immersion in diluted TDE (*n* = 1.46) and imaged them using a 1 -mm-thick fused-silica plate (*n* = 1.46) with a large 10 × 10 -cm viewing area (Fig. 3b; Supplementary Movie 5). High-magnification views of our data sets enabled unambiguous visualization of neuronal structures. We also cleared and labeled multiple mouse organs using the Ce3D protocol (*n* = 1.49) and imaged them in a single automated session using a customized six-well plate with a 0.5 -mm-thick PMMA bottom substrate (*n* = 1.49) (Fig. 3c; Supplementary Movie 6). Lymph nodes, heart, prostate, and lung tissues (1–4 -mm thick) were mounted in separate wells and imaged in toto for 3D visualization of immune cells in lymph nodes, the ventricles and valves within the heart, the glandular network within the prostate, and the bronchial tree within the lung^25^. The volume of these organs span a range of ~1 mm^3^ (lymph nodes)–100 mm^3^ (mouse heart), the latter being similar to a whole mouse brain, which is commonly imaged with LSFM systems. Note that the use of multi-well plates (e.g., six-well or 96-well plates) prevents contamination between specimens, which is particularly important for clinical assays and cell culture (a feature not easily achieved with other LSFM systems).

Expansion-based protocols provide a magnified view of structures that are otherwise too small to resolve with a given microscope^26–28^. To date, these expanded hydrogel specimens have consisted mostly of water, and therefore have a refractive index close to that of pure water (*n* = 1.33). While there are currently no glasses at this refractive index, fluoropolymers (i.e., Teflons), including fluorinated ethylene–propylene (FEP) and polytetrafluoroethylene (PTFE) possess a compatible refractive index (*n* = 1.34). These materials can be manufactured as thin sheets and stretched tight as drumhead surfaces that are ideal holders for expanded specimens. Using a customized drumhead, we imaged a 4 × expanded 250-μm-thick-kidney section (Fig. 3d; Supplementary Movies 7–9). After expansion, the physical size of the tissue was 2.1 × 3.2 × 0.1 cm. Representative zoomed-in views provide a highly detailed view of 3D structures, such as glomeruli, renal tubules, and blood vessels.

## Discussion

We have developed and characterized a multi-immersion OTLS microscope that enables high-throughput automated imaging of optically cleared specimens with an ease of use that should facilitate broader adoption of light-sheet-based 3D microscopy by both researchers and clinicians. Our system imposes minimal constraints on specimen size/shape and allows for fast and convenient mounting of multiple tissue specimens in individual wells. In particular, we demonstrate the ability to achieve sub-micron in-plane resolution at an imaging speed ~1 mm^3^ min^−1^ (per wavelength channel) with a maximum usable imaging depth of 0.5 cm and a lateral area of up to 10 × 10 cm. Both the system design and specimen holders can also be tailored and improved for specific research applications in future designs (see Supplementary Fig. 3, Supplementary Fig. 7, and Supplementary Discussion). Note that since the current OTLS system utilizes a stage-scanning approach, it is not ideal for imaging living organisms that move or change rapidly over time. Furthermore, due to the off-axis orientation of the OTLS beam paths, extremely high-NA objectives with short working distances are not easily incorporated. Therefore, OTLS microscopy is best-suited for convenient high-throughput imaging of large and/or multiple specimens at moderate resolution (~micron-scale) rather than extremely high-resolution (e.g., NA > 1.0) imaging of small tissue volumes. Finally, due to its open-top geometry, our system provides unsurpassed versatility to interface with a wide range of potential accessory technologies, such as microfluidic devices, single-cell electrophysiology, and micro-aspiration^29–32^.

## Methods

### Multi-immersion open-top light-sheet microscope

An optical schematic of the system is shown in Supplementary Fig. 8, and was modeled using commercially available ray-tracing software (ZEMAX LLC) (Supplementary Figs 9, 10, available as Supplementary ZEMAX Files). Illumination light is coupled into the system by a single-mode fiber with a numerical aperture of 0.12 from a four-channel digitally controlled laser package (Skyra, Cobolt Lasers). Light emanating from the fiber is collimated with a lens, L1 (AC1–128–019-A, *f* = 19 mm), and then expanded along one axis using a 3 × cylindrical telescope consisting of lenses, C1(ACY-254–50-A, Thorlabs, *f* = 50 mm) and C2 (ACY-254–150-A, Thorlabs, *f* = 150 mm) to provide multi-directional illumination (Supplementary Fig. 11)^33^. The resulting elliptical Gaussian beam is then relayed to the scanning galvanometer, GM (6210 H, Cambridge Technology) using lenses R1 (AC-254–100-A, Thorlabs, *f* = 100 mm) and R2 (AC-254–050-A, Thorlabs, *f* = 50 mm). The scanning mirror is driven by a sinusoidal voltage from a waveform generator (PCI-6115, National Instruments) at a frequency of 800 Hz. The sinusoidal waveform is mechanically more stable than a sawtooth waveform and increases the illumination intensity at the field edges, which partially offsets any vignetting due to the collection optics. The scanning beam is relayed to the back focal plan of the illumination objective (XLFLUOR340/4 × 0.28 NA, Olympus) using a scan lens, SL (CLS-SL, Thorlabs, *f* = 70 mm) and tube lens, TL1 (TTL200, Thorlabs, *f* = 200 mm). Finally, the elliptical beam travels through the plano-convex lens (LA4725, Thorlabs, *R* = 34.5 mm), immersion medium, holder, and finally specimen. During imaging, the open-top of the system is covered with a blackout covering (BT4, Thorlabs) to provide eye safety from the laser illumination and to reduce background from ambient lighting.

Fluorescence is collected by a multi-immersion objective (#54–10–12, Special Optics, distributed by Applied Scientific Instrumentation). This provides < 1 μm in-plane resolution for all immersion media (Supplementary Fig. 12). The fluorescence is filtered with a motorized filter wheel (FW102C, Thorlabs) with band-pass filters for the 405 -nm (FF02–447/60–25, Semrock), 488 -nm (FF03–525/50–25, Semrock), 561 -nm (FF01–618/50–25, Semrock), and 638 -nm (FF01–721/65–25, Semrock) excitation wavelengths. The filtered fluorescence is focused onto a 2048 × 2048 pixel sCMOS camera (ORCA-Flash4.0 V2, Hamamatsu) by a tube lens, TL2 (TTL165, Thorlabs, *f* = 165 mm). The tube lens provides a sampling of ~0.44 μm pixel^−1^ at *n* = 1.56 which satisfies the Nyquist criterion. This results in a horizontal field of view of ~0.9 mm over the 2048 pixels of the camera. The vertical field of view is reduced to 256 pixels (113μm) to closely match the depth of focus of the illumination light sheet (~110 μm). The 256 pixels are oriented parallel to the rolling shutter readout direction of the camera, which provides an exposure time of 1.25 ms and a framerate of 800 Hz. Similar to the dependence of the multi-immersion collection objective’s numerical aperture on refractive index, the magnification (and therefore pixel sampling) also varies with refractive index according to the following relationship: *M* = (165 mm/17.4 mm) × *n*. The sampling for each immersion medium used in this study are shown in Supplementary Table 1.

The maximum specimen thickness is limited by the usable working distance of the collection objective (0.5 cm) minus the thickness of the specimen holder. The immersion chamber requires ~ 300 mL of media to fill. When changing media, a motorized pipette is first used to remove all media, the chamber walls and optics are carefully cleaned with 70% ethanol, and the new media is then poured in— a process which in total takes ~30 min. The illumination objective, solid-immersion lens, and collection objective interface with the immersion chamber through customized aluminum mounts (Supplementary Fig. 13) which are available as Supplementary CAD Files.

Image strips are collected with a combination of stage-scanning and lateral/vertical tiling using a motorized XY stage and Z actuators (FTP-2050-XYZ, Applied Scientific Instrumentation, ASI). The stage-scanning firmware is used to send a TTL trigger signal from the XY stage to the sCMOS camera for reproducible start positioning (< 1 μm) of each image strip (Supplementary Fig. 14). The spatial interval between successive frames is set to ~0.31 μm, which, given the 800 Hz camera framerate, corresponds to a constant stage velocity of ~0.25 mm sec^−1^. For lateral tiling, an offset of 0.8 mm between adjacent image strips is used (~11% overlap). For vertical tiling, the 113-μm vertical field of view is oriented at 45 ° within the specimen, which corresponds to an image strip height of ~80 μm. Therefore, a vertical tiling offset of 70 μm is used (~12% overlap). The laser power is increased with depth per a user defined attenuation coefficient, *P* = *P*_0_ × exp(*z*/*μ*), to account for the attenuation of the illumination light sheet as it penetrates deeper into the specimen. The entire image acquisition is controlled by a custom LabVIEW (National Instruments) program. As shown in Supplementary Fig. 15, the program consists of a series of nested loops for imaging multiple specimens, collecting multiple color channels, and lateral/vertical tiling. A complete list of components is available in Supplementary Table 3.

### Computer hardware

During acquisition, the images are collected at the maximum data-transfer rate (~800 MB sec^−1^) by a dedicated workstation (Precision Tower 5810, Dell) equipped with a CameraLink interface (Firebird PCI Express, Active Silicon). The data are streamed in real-time using the proprietary DCIMG Hamamatsu format to a mapped network drive located on an in-lab server (X11-DPG-QT, SuperMicro) running 64-bit Windows Server, equipped with 384 GB RAM and TitanXP (NVIDIA) and Quadro P6000 (NVIDIA) GPUs. The server contains two high-speed RAID0 storage arrays of 4 × 2.0 TB SSDs, as well as a larger direct-attached RAID6 storage array with 15 × 8.0 TB HDDs. All RAID arrays are hardware based, the RAID0 arrays are controlled by an internal 8-port controller (LSI MegaRaid 9361–8i 1 GB cache) and the RAID6 array is controlled by an external 8-port controller (LSI MegaRaid 9380–8e 1 GB cache). Both the server and acquisition workstation are equipped with 10 G SFP + network cards, jumbo frames, and parallel send/receive processes matched to the number of computing cores on the workstation (8 physical cores) and server (16 physical cores), which reliably enables > 1.0 GB sec^−1^ network-transfer speeds (to accommodate the data-transfer rate of the sCMOS camera and enable simultaneous data-processing routines). The hardware setup is shown in Supplementary Fig. 16. The complete hardware configuration is listed in Supplementary Table 4.

### Data processing and visualization

Collected data sets undergo a Python pre-processing routine before being visualized in 2D and 3D by several open-source and commercial packages. Each image strip is stored in a single DCIMG file. These DCIMG files are read into RAM by a DLL compiled using the Hamamatsu DCIMG software development kit (SDK) and first de-skewed at 45 ° By precisely setting the interval between successive frames, the de-skewing is quickly performed by simply shifting each plane of pixels in the image strip by an integer pixel offset (Supplementary Fig. 17). This operation is extremely fast compared with alternative de-skewing approaches using computationally expensive affine transformations, but can introduce artifacts if an incorrect scan interval is used. The data are then written from RAM to disk using the Hierarchical Data Format (HDF5) with the metadata and XML file structured for subsequent analysis using BigStitcher^34^. A custom HDF5 compression filter (B3D) is used with default parameters to provide ~10 × compression, which is within the noise limit of the sCMOS camera^35^. This pre-processing routine is applied to all DCIMG files, ultimately resulting in a single HDF5/XML file for BigStitcher. The starting position of each image strip is first set in BigStitcher using the expected experimental stage coordinates. These starting coordinates are valid due to the effective immobilization of the specimens by gravity and, in some cases, gentle pressure applied on the specimen during imaging. The fine alignment of all image strips was then performed in BigStitcher, and finally fused to disk in either TIFF or HDF5 file formats. The resulting TIFF and HDF5 files are then visualized using open-source and commercial packages, including ImageJ, BigDataViewer, Aivia (DRVision), and Imaris (Bitplane)^36,37^. To optionally provide false-colored pseudo-H&E histology images, a Beer-Lambert coloring algorithm is applied using a Python script^38^. The entire processing pipeline and representative processing times are shown in Supplementary Fig. 18. For a representative 1-TB data set with two channels and ten imaging tiles, image acquisition requires ~20 min, shearing requires ~20 min, conversion from DCIMG to a compressed HDF5 file requires ~3 h, tile alignment requires ~5 min, and 3D fusion (if desired) requires 12–24 h. All processing files are available as Supplementary Code. Supplementary Data and instructions for installing and using the computer code are also available at https://figshare.com/articles/Supplementary_Data/7685597.

### Specimen holders

All holders were attached to the motorized XY stage using custom machined aluminum adapters plates (HILLTOP21). For the mouse brain slices, a 1-mm-thick fused silica window (Esco Optics) with a 10 × 10-cm cross-section was attached to a custom adapter plate using UV-curing glue (NOA60, Norland Products) (Supplementary Fig. 19). Mouse organs cleared using Ce3D were imaged on a customized six-well plate. The bottom of a conventional polystyrene six-well plate (Cat:CLS3506, Sigma-Aldrich) was removed using an in-house desktop mill (OtherMill, Bantam Tools) with a 1/8 inch flat-end drill bit. The drill speed was set to 12,000 rpm, feed rate to 600 mm min^−1^, and plunge rate to 12 mm min^−1^. The bottom was then replaced by a 0.5 -mm-thick PMMA plate (Goodfellow USA) attached using UV-curing glue (NOA60, Norland Products) (Supplementary Fig. 20). For the expanded kidney specimen, a custom drumhead was fabricated and adapted for mounting to the microscope. The drumhead tightens a 0.1 -mm-thick FEP film over an extruded opening, which is ideal for OTLS imaging of expanded specimens (Supplementary Fig. 21). To overcome the hydrophobic nature of the FEP films (which cause drifting of expanded specimens), the upper surface of the FEP films were treated with 0.1% (w/v) poly-lysine (Cat:P8920, Sigma-Aldrich) for charged-based adhesion of specimens to the FEP surface. For the human prostate biopsies, HIVEX lens blanks (Conant Optical) were purchased and custom machined using an in-house desktop mill (OtherMill, Bantam Tools) (Supplementary Fig. 22). The 1/8 inch, 1/16 inch, and 1/32 inch drill bits were used with the drill speed set to 12,000 rpm, feed rate set to 500 mm min^−1^, and plunge rate set to 10 mm min^−1^. CAD files for all specimen holders are available as Supplementary CAD Files. The system can also be used as a whole-slide scanner for conventional fluorescently labeled histology slides using a commercially available slide holder (MLS203-P2, Thorlabs) (Supplementary Fig. 23). All specimen holders can be re-used for multiple imaging experiments. Between each experiment, specimen holders are removed and cleaned with 70% ethanol before reuse. Dispersion curves for the various holder materials and clearing reagent combinations are shown in Supplementary Fig. 24.

### Optical simulations

Optical simulations were performed using commercially available ray-tracing software (ZEMAX, LLC) with a blackbox model of the multi-immersion objective (provided by the manufacturer, Special Optics). For the simulations shown in Fig. 2, the base refractive index of the immersion medium and specimen was assumed to be *n* = 1.45, and the optical path difference was varied. For all scenarios, the imaging depth was set to 1 mm, and the PSF was measured at the center of the imaging field of view, as well as at the field edges (shown in Supplementary Fig. 10). The same relationship between Strehl Ratio and optical path difference was observed for other base refractive indices and imaging depths, under the assumption that the optical properties of the immersion medium and specimen were the same. These same parameters (with reduced collection NAs) were used to generate the data shown in Supplementary Fig. 6. The ZEMAX files for the OTLS system are available as Supplementary ZEMAX Files.

### Collection and processing of mouse brain slices

A mouse of line Sst-IRES-Cre;Ai139(TIT2L-GFP-ICL-TPT), characterized previously^39^, was used for imaging experiments. Genotyping confirmed expression of Cre and tdTomato for this individual. The mouse was killed at age P96 by transcardial perfusion with 4% paraformaldehyde. The brain was dissected and post fixed in 4% paraformaldehyde at room temperature for 3–6 h followed by overnight fixation at 4 ° C. The brain was rinsed with 1 × PBS and stored in 1 × PBS with 0.1% sodium azide prior (Cat:S2002, Sigma-Aldrich) prior to sectioning. 200-µm-thick cortical sections were cut on a vibratome and stored in 1 × PBS. Prior to OTLS imaging, brain slices were incubated in a mixture of 68% 2,2′-thiodiethanol (TDE) (Cat:166782, Sigma-Aldrich) and 32% 1 × PBS for clearing. The refractive index of the solution (*n* ~ 1.46) was verified using a refractometer (PA202, Misco). Procedures involving mice were complied with ethical regulations and were approved by the Institutional Animal Care and Use Committee of the Allen Institute for Brain Science in accordance with NIH guidelines.

### Collection and processing of whole mouse organs

The lung, heart, prostate, and lymph nodes were collected from a CD11-YFP, Actin-dsRed expressing mouse. Tissues were fixed for 24 h at 4 °C in 1 part fixative (Cat:554655, BD Biosciences) and 2 parts 1 × PBS and incubated in blocking buffer for 24 h at 37 °C. The buffer consisted of 30 mL of Tris (Cat:252859, Sigma-Aldrich), 0.3 mL of NMS (Cat:SML1128, Sigma-Aldrich), 0.3 mL of BSA (Cat:A2058, Sigma-Aldrich), and 0.09 mL of TritonX100 (Cat:T8787, Sigma-Aldrich). Lymph nodes were stained for 4 days at 37 °C in 400 μL of blocking buffer, 2 μL of CD3-BV421 (Cat: 100228, BioLegend) (1:200 dilution), and 2 μL of B220-e660 (Cat: 50–0452–82, Thermo-Fisher) (1:200 dilution). Lung tissue was stained for 3 days at 37 °C in 500 μL of blocking buffer and 2.5 μL of Epcam-APC (Cat: 17–5791–82, Thermo-Fisher) (1:200 dilution). Heart tissue was stained for 1 day with 1 mM DRAQ5. Prostate tissue was incubated with fluorophore-conjugated anti-CK8–18 (Cat:MS743S0, Thermo-Fisher) conjugated to Alexa-Fluor 488 (Cat: A20181, Invitrogen) (1:100 dilution) in 1 × PBS, 1% nonfat dry milk, and 0.2% Triton X-100 at 37 °C for 7 days with gentle agitation. All tissues were then cleared with the Ce3D solution, consisting of 14 mL of 40% N-methyl-acetamide (Cat: M26305, Sigma-Aldrich), 25 μL of Triton X-100 (Cat: T8787, Sigma-Aldrich), 20 g of Histodenz (Cat: D2158, Sigma-Aldrich), and 125 μL of thioglycerol (Cat: 88640, Sigma-Aldrich) for 1 day at room temperature. Procedures involving mice complied with ethical regulations and were approved by the Institutional Animal Care and Use Committee of the University of Washington in accordance with NIH guidelines.

### Collection and processing of expanded mouse kidney

In total, 4% PFA-fixed mouse kidney was sliced to 200 μm and processed using an expansion microscopy protocol^40^. The tissue was first incubated in blocking/permeabilization buffer for 6 h at 4 ° C. Primary antibodies goat anti-podocalyxin (Cat: AF1556, R&D Sys. Inc.) and rabbit anti-collagen IV (Cat: ab6586, Abcam) were diluted 1:50 with blocking/permeabilization buffer and used to stain the tissue for 2 days at 4 °C. The tissue was then washed with 1 × PBS three times at room temperature (1 h each). Fluorescently labeled secondary antibodies, Alexa 488-conjugated WGA (Cat:W11261, Thermo-Fisher), diluted 1:25, and Hoechst 33342 were then diluted in blocking/permeabilization buffer to stain the tissue for 2 days at 4 °C. The tissue was washed with 1 × PBS three times at room temperature (1 h each) followed by incubating in 1 mM MA-NHS (Cat:730300, Sigma-Aldrich) for 1 h at room temperature. The tissue was then incubated in monomer solution for 1 h at 4  °C and then gelled in a humidified environment at 37 °C for 2 h. Excess gel was removed and the specimen was digested by proteinase K (Cat:EO0491, Thermo-Fisher) at 37 ° C for 2 days and then collagenase (Cat: C7926, Sigma-Aldrich) at 37 °C for 2 days refreshing the solution daily. After digestion, the specimen was incubated in deionized water for at least 2 h and the expansion factor was determined through measuring the dimensions of the gel. The expanded specimen was mounted on poly-lysine-coated film for imaging. Procedures involving mice complied with ethical regulations and were approved by the Institutional Animal Care and Use Committee of the University of Washington in accordance with NIH guidelines.

### Collection and processing of human prostate biopsies

All specimens were obtained from an IRB-approved genitourinary biorepository with patient consent. Core-needle biopsy specimens were obtained from fresh ex vivo prostatectomy specimens using an 18-gauge (~1 -mm inner diameter) needle biopsy device (Bard Max Core, Bard Biopsy). The biopsy was immediately placed in 10% neutral buffered formalin, where it was maintained at room temperature for 24 h. In contrast to mouse tissues, we found that human tissues require more aggressive solvent-clearing approaches. Due to its clearing efficacy and non-toxic nature, we used ECi-clearing, which we observed does not interfere with downstream histology or immunohistochemistry (Supplementary Fig. 25).

Biopsies were then washed in 1 × PBS with 0.1% Triton X-100 (Cat: T8787, Sigma-Aldrich), and each biopsy was stained for 4 h in a 1:2000 dilution of TO-PRO3 Iodide (Cat:T3605, Thermo-Fisher) at room temperature with light shaking. Each biopsy was then dehydrated in ethanol for with 25/75, 50/50, 75/25, and 100/0 grades. The dehydration time for each grade was 1 h, and the 100% ethanol grade was performed twice to ensure removal of any excess water. Biopsies were then stained in 1:2000 dilution of Eosin-Y (Cat:3801615, Leica Biosystems) for 4 h at room temperature with light shaking. Finally, biopsies were cleared in ethyl-cinnamate (Cat: 112372, Sigma-Aldrich) for 1 h. Biopsy #12 was stained with anti-CK8. The biopsy issue was incubated simultaneously with fluorophore-conjugated anti-CK8–18 (Cat:MS743S0, Thermo-Fisher) conjugated to Alexa-Fluor 488 (Cat:A20181, Invitrogen) (1:100 dilution) in 1 × PBS, 1% nonfat dry milk, and 0.2% Triton X-100 at 37 °C for 7 days with gentle agitation. All human prostate biopsies were obtained prospectively from the University of Washington Genitourinary Biorepository with patient consent. Approval was obtained from the University of Washington Institutional Review Board.

### Reporting summary

Further information on research design is available in the Nature Research Reporting Summary linked to this article.

## Supplementary information

Supplementary Information

Description of Additional Supplementary Files

Supplementary Movie 1

Supplementary Movie 2

Supplementary Movie 3

Supplementary Movie 4

Supplementary Movie 5

Supplementary Movie 6

Supplementary Movie 7

Supplementary Movie 8

Supplementary Movie 9

Reporting Summary

## Data Availability

All raw and processed data generated in this work, including the images provided in the manuscript and supplementary material are available from the authors upon request. The customized CAD and ZEMAX files are available as Supplementary CAD Files and Supplementary ZEMAX Files at https://figshare.com/articles/Supplementary_Data/7685597.

## Code availability

The computer code used to acquire, process, and generate the images in this study is open-source and available as Supplementary Code Files. Supplementary Data and instructions for installing and using the computer code are available at https://figshare.com/articles/Supplementary_Data/7685597.
