# Supplementary material for: Multi-immersion open-top light-sheet microscope for high-throughput imaging of cleared tissues
Source: Nat Commun. 2019 Jul 4;10:2781. doi: 10.1038/s41467-019-10534-0 (PMC6609674; doi:10.1038/s41467-019-10534-0)
Supplement: Supplementary file 1 — Supplementary Information [file 41467_2019_10534_MOESM1_ESM.pdf]

Supplementary Information

Multi-immersion open-top light-sheet microscope for high-throughput imaging of cleared tissues

Glaser *et al.*

Supplementary Discussion

Refractive index considerations for various clearing protocols

Precise refractive index matching of the immersion medium, holder, and specimen is necessary for aberration-free imaging with the open-top architecture. This refractive index value is dictated by the clearing protocol used on the specimen, and spans a wide range (n = 1.33 – 1.56). These protocols can be categorized into aqueous, solvent, and expansion protocols [1-23].

Aqueous-based protocols involve optional lipid removal using detergents, followed by immersion in mixtures of a single or multiple water-soluble reagents. Because these mixtures contain some dilution of water, their exact refractive index value is difficult to control precisely (for the purposes of matching the index of the specimen holder material). For example, when initially clearing a specimen, the water content of the specimen will homogenize with the volume of refractive index matching media, potentially leading to a slightly different final refractive index than intended. Therefore, several successive refractive index matching immersion steps are necessary to achieve the intended specimen refractive index. In addition, evaporation of water content from the specimen can lead to a change in refractive index over time. To mitigate this, specimens should be covered during imaging.

Solvent-based protocols involve dehydration of tissue specimens, and replace water content with organic reagents of a higher refractive index. Unlike aqueous protocols, the refractive index of solvent-cleared specimens is easier to control. However, similar to the issue of water content for aqueous-based protocols, the alcohol used for dehydration will homogenize with the final organic reagent. To achieve the desired final refractive index, we found that specimens should be cleared multiple times in the final organic reagent, or in a large enough volume of reagent such that the volume of alcohol within the specimen has a negligible effect on the specimen's final refractive index.

Finally, expansion-based protocols involve several steps which physically magnify specimens by swelling a hydrogel. To date, these protocols have all used water (n = 1.33) for the final swelling and expansion steps. Therefore, the refractive index of expanded specimens is easy to control. However, care must be taken to cover the gels during imaging and prevent them from drying out.

Material selection and dispersion

As mentioned in the main manuscript, diffraction-limited imaging performance is only achieved for optical

path differences of $\Delta n \times t < 0.002$ mm. However, the refractive index of materials and clearing reagents all vary with wavelength (i.e., dispersion) (see **Supplementary Figure 24**). The dispersion of glasses are well characterized and available from manufacturers and online resources. However, the dispersion of monomers and polymers are not as well characterized and more difficult to obtain. Finally, the dispersion of many clearing reagents has not been characterized. While the dispersion of more common reagents (e.g., water, TDE, DMSO, and Glycerol) is available, the increasing complexity of clearing cocktails, particularly for aqueous protocols, makes it difficult to determine the final mixture's refractive index. For example, the latest CUBIC clearing protocol uses several reagents including antipyrine/N-methylnicotinamide, all of which have not been characterized [24]. Therefore, future design and optimization of OTLS-based systems will require more precise characterization of the dispersion of various clearing reagents and holder materials.

Objective design for open-top imaging

In addition to index matching, the performance of the OTLS system depends critically on the choice of microscope objectives. For the current system, a multi-immersion objective designed by ASI / Special Optics was utilized. This particular objective has a specific numerical aperture (NA) and working distance (WD). The WD dictates the maximum imaging depth (h) of our system (see **Supplementary Figure 3**). However, future systems requiring either lower or higher resolution (with a resulting trade-off in field of view), and more or less imaging depth, could be designed around a different objective. For a given NA, the maximum imaging depth can be extended by increasing the WD of the objective, which usually increases the physical size of the objective and the diameter of the pupil near the back focal plane of the objective. The limiting factor to increasing the size of the objective would be cost and compatibility with other optical and mechanical components. The relationship between the NA and h is plotted in **Supplementary Figure 3**. Note that in practice, h will be less than the values shown since the mechanical housing of most objectives will protrude beyond the front element of the objective, which reduces the usable working distance of the objective.

Future improvements for open-top imaging

In addition to alternative collection objectives with varying NA and WD, the optical architecture of the

system can also be improved in the future. Unlike living specimens, cleared specimens are stationary, and therefore the current system is able to use extensive image tiling to stitch together large 3D volumes. While tiling to some degree is unavoidable, the amount of vertical tiling can be reduced in a future design by utilizing the full 2048 pixels of the camera, rather than the current 256 pixels (which is limited by the Rayleigh range of the Gaussian illumination beam). While the imaging speed remains the same (i.e. the camera operates at 800 Hz for 256 pixels, and only 100 Hz for 2048 pixels), capturing 2048 pixels directly on the camera chip would reduce the number of vertical tiles by a factor of 8. This would result in a slight increase in imaging speed since each vertical tile overlaps by a small amount with adjacent tiles. However, the final image-fusion step (if desired to create a full volume rendering of the data) is mainly limited by the time required to read and write the data from/to the disk (rather than stitching/blending the seams between tiles). Therefore, the speedup in processing time is marginal.

Generating a light sheet with a longer depth-of-focus to cover the full 2048 pixels of the camera could be achieved by using a propagation-invariant beam (e.g. Bessel or Airy) or a lower-NA Gaussian light sheet. Alternatively, an attractive (albeit technological complex) option would be the use of an axially swept light sheet [25]. This axially sweeping architecture would provide an excellent balance of speed, contrast, and resolution. In addition, if the illumination NA is matched to the collection NA, isotropic resolution can be achieved without the extra computational complexities of dual-view imaging (as with the diSPIM system) [26].

Another challenge for all 3D imaging systems is achieving an optimal balance between imaging resolution and imaging speed (volume per unit time). A doubling in resolution results in an 8× decrease in imaging speed. Therefore, it is usually most ideal to image with the lowest resolution to address a given biomedical application. Difficulties arise when the goal is to identify and investigate small (high-resolution) structures that are sparsely distributed at unknown locations within a large specimen. Therefore, future improvements can also include the ability to perform multi-resolution imaging, either with a single high space-bandwidth objective (i.e., a large field of view and large NA), or multiple objectives of varying magnification and field of view. This would enable rapid screening of specimens to identify regions of interest at lower resolution, followed by higher-magnification imaging of these regions for additional insights and/or confirmation that the microstructures of interest have been identified.

Finally, orienting the illumination and collection objectives at oblique (e.g. 45-deg) angles enables laterally unconstrained imaging at the cost of a reduced usable working distance for a given set of microscope objectives. To maintain the full usable working distance of a collection objective, the light-sheet theta microscopy (LSTM) could be adapted to the open-top architecture [27]. This adaptation would also lessen the index matching requirement for the collection objective (oriented at 90 deg to the specimen holder, rather than at 45 deg), although precise index matching would still be required for the off-axis illumination objectives. While this non-orthogonal arrangement maintains the full working distance of the collection objective, it would not be able to provide isotropic resolution. Therefore, future improvements to the system will all come with inherent trade-offs, and should be tailored to a given biomedical application. A summary is given in **Supplementary Figure 7**.

Supplementary Figures

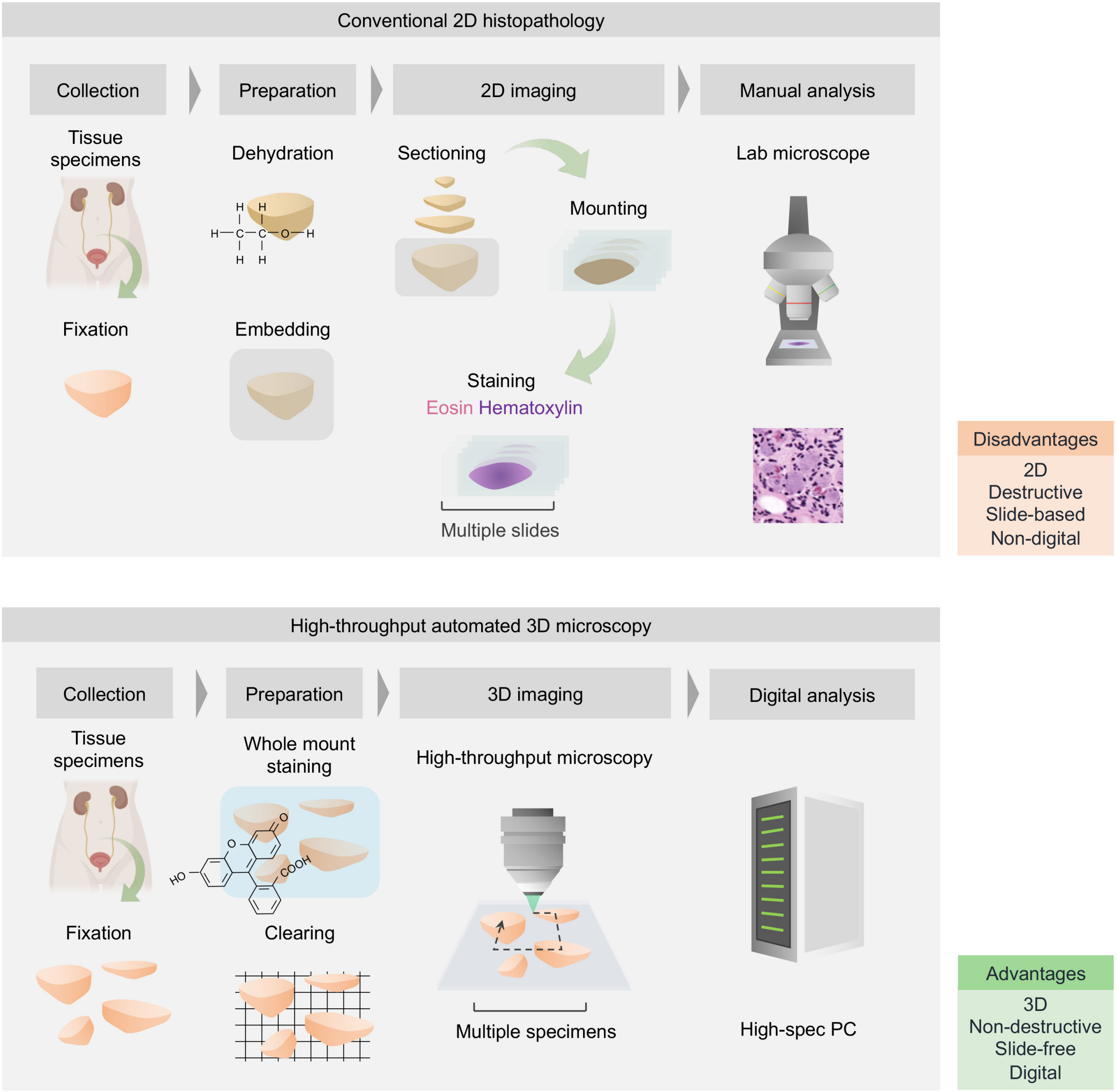

Supplementary Figure 1. Comparison of 2D histopathology and 3D microscopy. The conventional histopathology protocol requires specimens to be fixed, dehydrated, embedded in paraffin wax, physically sectioned into thin slices, and stained with hematoxylin and eosin (H&E). Slides are then manually analyzed with a conventional laboratory microscope. With the multi-immersion OTLS system, multiple specimens are collected, fixed, stained with fluorescent dyes, and cleared (using an aqueous, solvent, or expansion protocol). Multiple specimens are then mounted on the system and imaged in an automated manner. The resulting 3D data is then digitally analyzed using a computer. In comparison to conventional histopathology, this workflow is 3D, non-destructive, slide-free, and inherently digital.

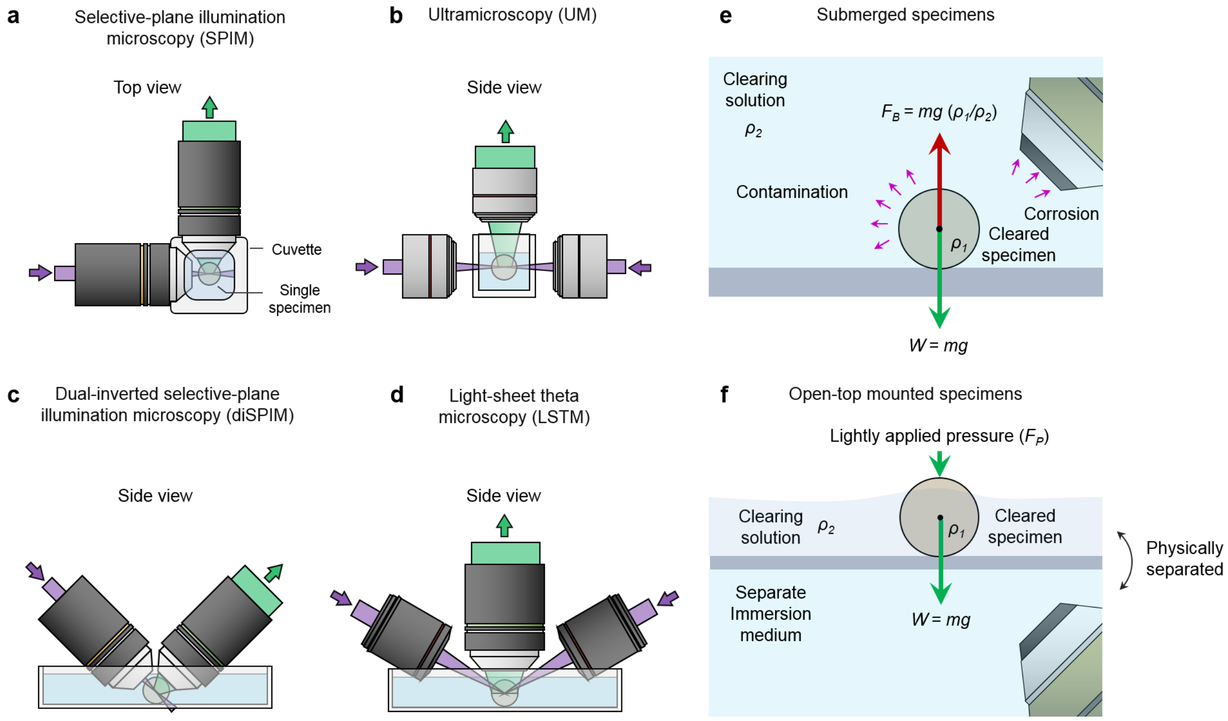

Supplementary Figure 2. Alternative LSFM architectures. (a-d) The geometry of SPIM, UM, diSPIM, and LSTM systems are shown. SPIM and UM systems place constraints on specimen size, shape, and number, which limit the ability to perform automated high-throughput imaging. **(e)** diSPIM and LSTM systems require specimens to be submerged in a large liquid reservoir. In comparison to open-top mounted specimens, this has several disadvantages **(f)**. The imaging objectives and specimen both share a common liquid reservoir, which results in inter-specimen contamination and dilution of reagents. In addition, these systems are unable to image tissues cleared with certain highly corrosive organic solvents (e.g., dibenzyl-ether, benzyl-alcohol, and benzyl-benzoate) without the use of specialized immersion objectives. Finally, the cleared tissues equilibrate to a similar density as the surrounding liquid reservoir, causing them to float and drift. This creates logistical challenges in terms of mounting and immobilizing specimens for large-volume imaging experiments. See the **Supplementary Discussion**.

a Simplified model

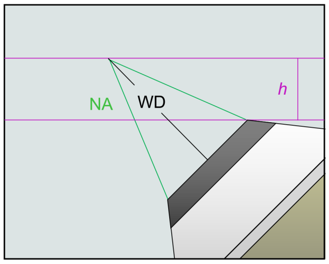

b Additional factors considered

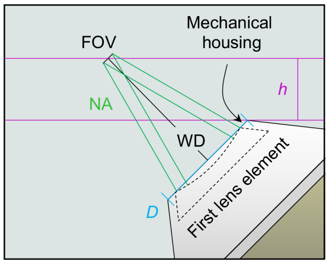

Variables
h – Maximum theoretical imaging depth
NA – Numerical aperture
D – Mechanical diameter of objective tip
WD – Working distance
FOV – Field of view

Equations
h = WD / √2 x (1– NA)
h_{actual} = WD / √2 x (1– sin(tan^{-1}(D/(2 xWD))))
BFP diameter = 2 x EFL x NA

c Objectives used in current OTLS system

Olympus XLFLUOR 340/4X	
NA	0.28
WD	29.5 mm
EFL	45 mm
D	35 mm
FOV	5.5 mm
BFP diameter	25.2 mm
h	~ 15 mm
h_{actual}	~ 5.7 mm

ASI / Special Optics objective	
NA	0.276
WD	12 mm
EFL	17.4 mm
D	9.8 mm
FOV	1.2 mm
BFP diameter	9.6 mm
h	~ 6.1 mm
h_{actual}	~ 5.1 mm

Supplementary Figure 3. Objective design for open-top imaging. (a) Simplified model depicting an objective oriented at 45 deg for open-top imaging. The numerical aperture (NA), working distance (WD), and maximum theoretical clearance (*h*), are shown. **(b)** Model with additional factors considered, including the field of view (FOV) and mechanical housing of the objective. The variables and equations for the clearance are shown below. The specifications for both objectives used in the current OTLS system are shown in **(c)**.

	Maximum specimen size	Isotropic resolution	Multi-specimen imaging	Usable working distance
**a** Ultramicroscopy (UM) 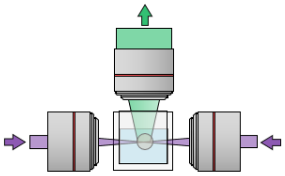	Imaging is constrained laterally by specimen holder and microscope architecture	Isotropic resolution not currently implemented	Logistically difficult to perform multi-specimen imaging due to issues associated with placing specimens in a large liquid reservoir.	Full working distance remains usable
**b** Dual-inverted selective-plane illumination microscopy (diSPIM) 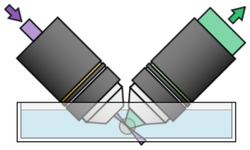	Off-axis objectives enable laterally unconstrained imaging	Dual-view imaging and deconvolution enable isotropic resolution	Logistically difficult to perform multi-specimen imaging due to issues associated with placing specimens in a large liquid reservoir.	Off-axis objectives lead to a reduction in the usable working distance
**c** Light-sheet theta microscopy (LSTM) 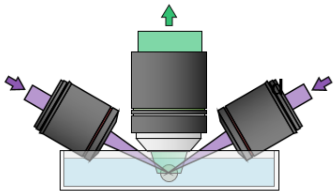	Lateral and axial scanning deliver of light sheet to collection objective focal plane enables laterally unconstrained imaging	Isotropic resolution not currently possible due to non-orthogonal arrangement	Logistically difficult to perform multi-specimen imaging due to issues associated with placing specimens in a large liquid reservoir.	Full working distance remains usable
**d** Open-top light-sheet microscopy (OTLS) 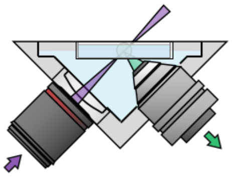	Off-axis objectives enable laterally unconstrained imaging	Isotropic resolution not implemented	Open-top geometry enables simpler and more robust specimen mounting which enables multi-specimen imaging	Off-axis objectives lead to a reduction in the usable working distance

Supplementary Figure 4. Comparison of cleared tissue LSFM systems. (a) UM is constrained laterally, does not currently offer isotropic resolution, is logistically difficult to use for multi-specimen imaging, but enables full use of the collection objective's working distance. **(b)** diSPIM is laterally unconstrained and able to achieve isotropic resolution (albeit with increased complexity in terms of hardware and computation), but is logistically difficult to use for multi-specimen imaging and has a reduced usable working distance. **(c)** LSTM is laterally unconstrained and is able to utilize the full working distance of the collection objective, but does not provide isotropic resolution. It is also logistically difficult to use for multi-specimen imaging. **(d)** OTLS is laterally unconstrained and enables simpler and more robust specimen mounting for multi-specimen imaging, but has a reduced usable working distance and does not currently provide isotropic resolution (though this is possible in the future, see the **Supplementary Discussion and Supplementary Fig. 7**).

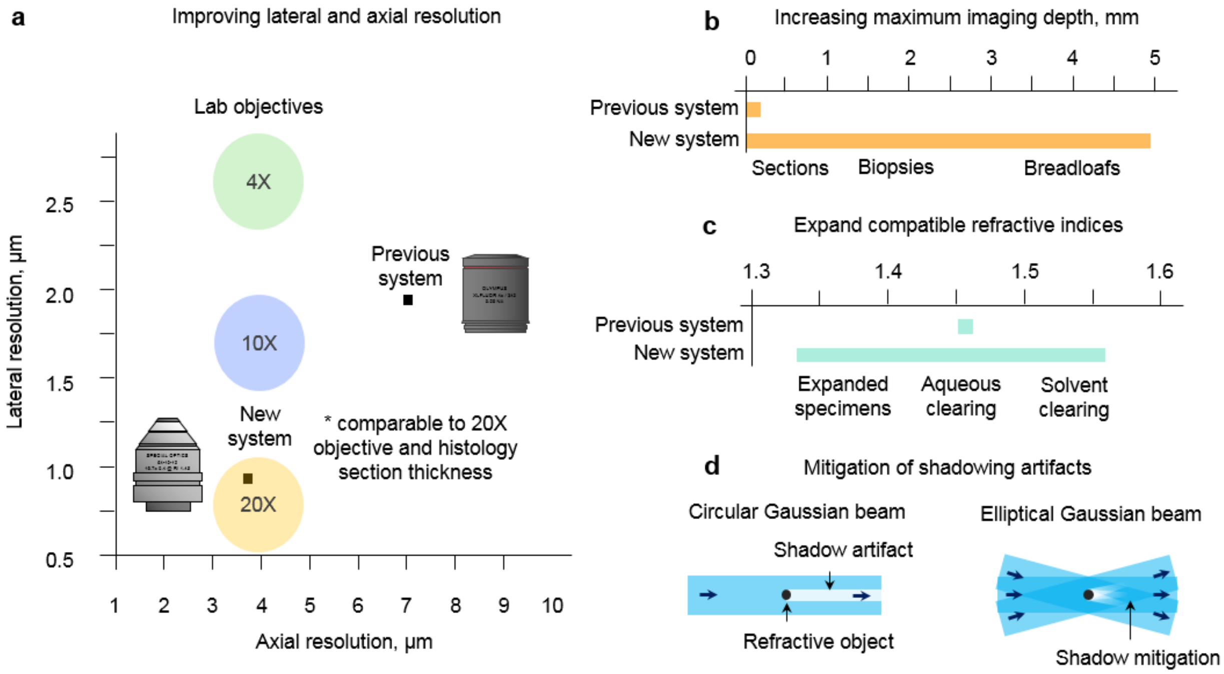

Supplementary Figure 5. System improvements. In comparison to a previous prototype system [28], our new OTLS system exhibits **(a)** improved axial and lateral resolution (an order-of-magnitude smaller focal volume), **(b)** ~20X greater imaging depth, **(c)** multi-immersion capabilities (n = 1.33 – 1.56), and **(d)** shadow mitigation.

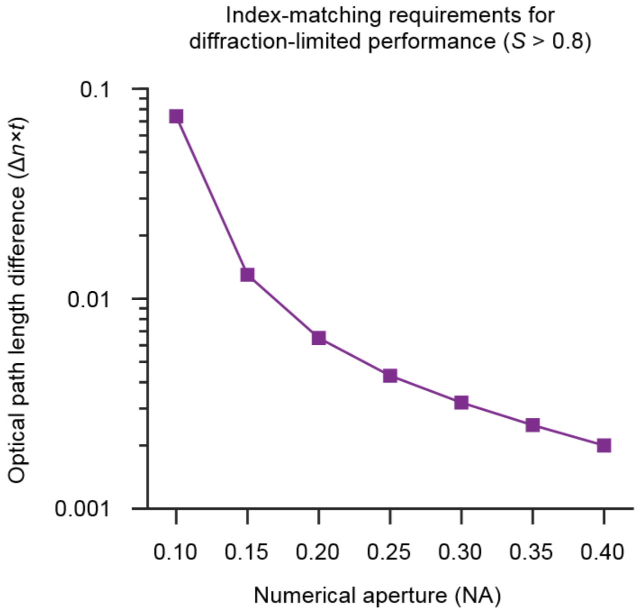

Supplementary Figure 6. Index matching requirements as a function of numerical aperture. To expand upon the results in **Figure 2**, the index matching requirement for diffraction-limited performance ($S > 0.8$) is plotted as a function of numerical aperture, in the range of 0.10 – 0.40.

	Usable working distance	Isotropic resolution
Open-top light-sheet theta microscopy 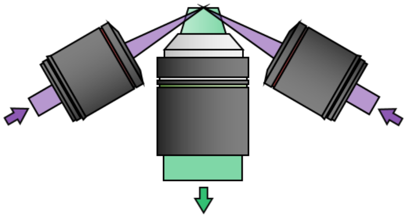	Full working distance remains usable	Isotropic resolution not currently implemented
Open-top axially-swept light-sheet microscopy 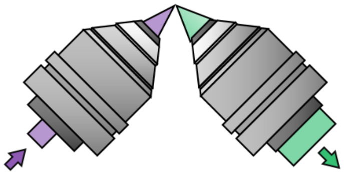	Off-axis orientation of objectives leads to a reduction in the usable working distance	Axially sweeping enables deconvolution-free isotropic resolution

Supplementary Figure 7. Future OTLS improvements. In future designs, the OTLS concept can be combined with light-sheet theta microscopy (to enable use of the full working distance of the collection objective), or axially-swept light-sheet microscopy (to enable deconvolution-free isotropic resolution).

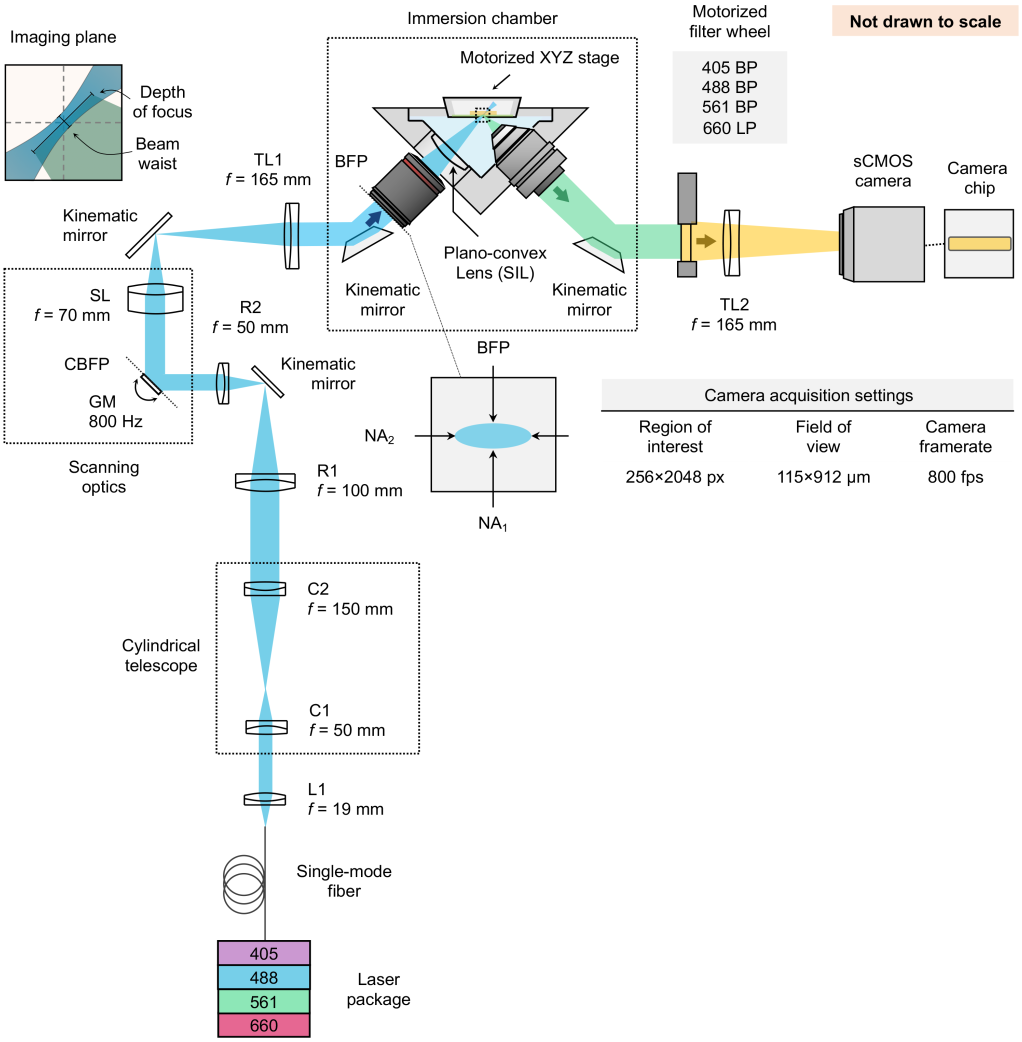

Supplementary Figure 8. Optical layout. Illumination light is coupled into the system by a single-mode fiber with a numerical aperture of 0.12 from a four-channel laser package, collimated with a lens, L1 (*f* = 19 mm), and then expanded along one axis using a 3× cylindrical telescope consisting of lenses, C1(*f* = 50 mm) and C2 (*f* = 150 mm) to provide multi-directional illumination. The resulting elliptical Gaussian beam is then relayed to the scanning galvanometer, GM (6210H, Cambridge Technology) using lenses R1 (*f* = 100 mm) and R2 (*f* = 50 mm). The scanning mirror is driven by a sinusoidal voltage from a waveform generator (PCI-6115, National Instruments) at a frequency of 800 Hz. The scanning beam is relayed to the back focal plan of the illumination objective (XLFLUOR340/4×, Olympus) using a scan lens, SL (*f* = 70 mm) and tube lens, TL1 (*f* = 165 mm). Finally, the elliptical beam travels through a plano-convex lens (*R* = 34.5 mm), immersion media, holder, and finally specimen. Fluorescence is collected by a multi-immersion objective (#54-10-12, Applied Scientific Instrumentation - ASI) which provides <1 µm in-plane resolution for all immersion media and filtered with a motorized filter wheel with band-pass filters for the 405 nm, 488 nm, 561 nm, and 638 nm excitation wavelengths. The filtered fluorescence is focused onto a 2048×2048 pixel sCMOS camera by a tube lens, TL2 (*f* = 165 mm). The tube lens provides a Nyquist sampling of ~0.44 µm/pixel (at *n* = 1.56), which provides a horizontal field of view of ~0.9 mm over the 2048 pixels of the camera. The vertical field of view is reduced to 256 pixels (113 µm) closely match the depth of focus of the illumination light sheet (~110 µm). The 256 pixels are oriented parallel to the rolling shutter readout, which provides an exposure time of 1.25 ms and a framerate of 800 Hz.

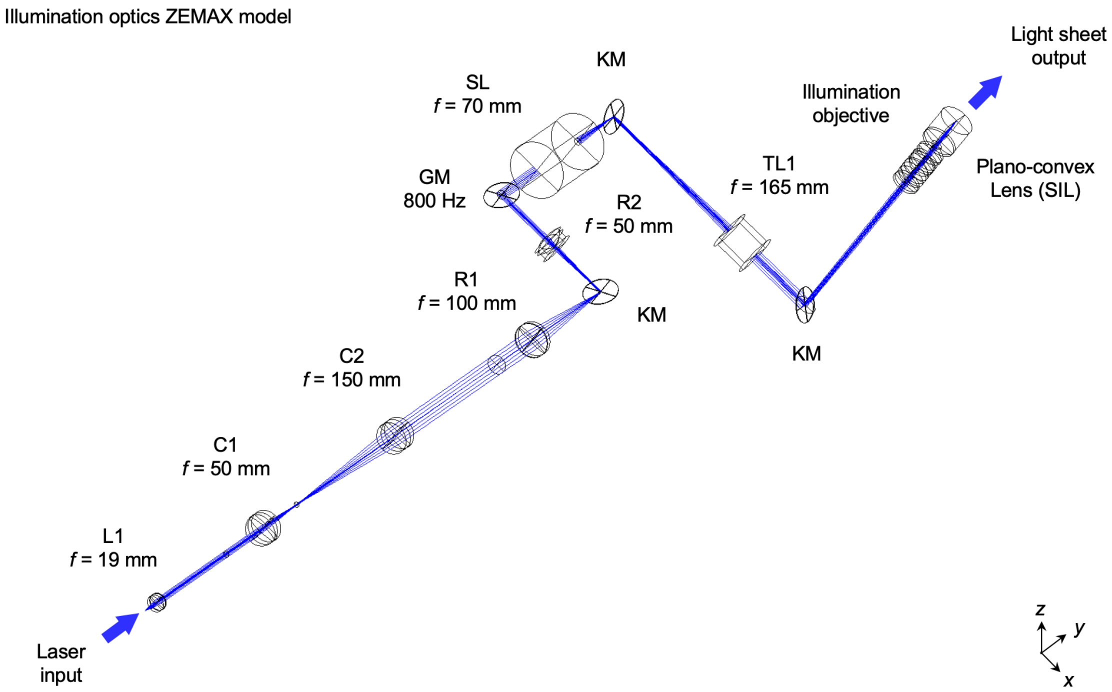

Supplementary Figure 9. Illumination optics model. 3D view of the illumination model, provided in **Supplementary ZEMAX files**. The illumination objective was modeled based on a patent [29]. All other components were modeled using manufacturer provided ZEMAX lens and blackbox files.

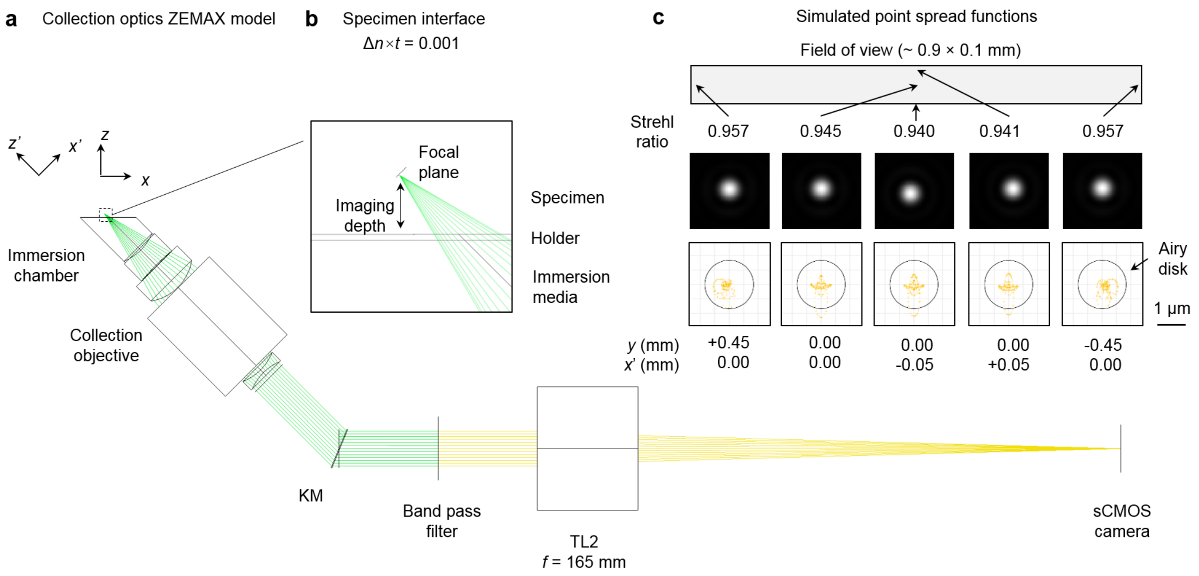

Supplementary Figure 10. Collection optics model. (a) ZEMAX model of the collection optics, provided in **Supplementary ZEMAX files**. A zoom-in of the specimen interface is shown in **(b)**. A representative detector spot diagram (ray-tracing) and simulated PSF (diffraction theory) are shown in **(c)**. This model file was used to generate the plot of Strehl Ratio (*S*) as a function of optical path difference in **Figure 2** of the main manuscript.

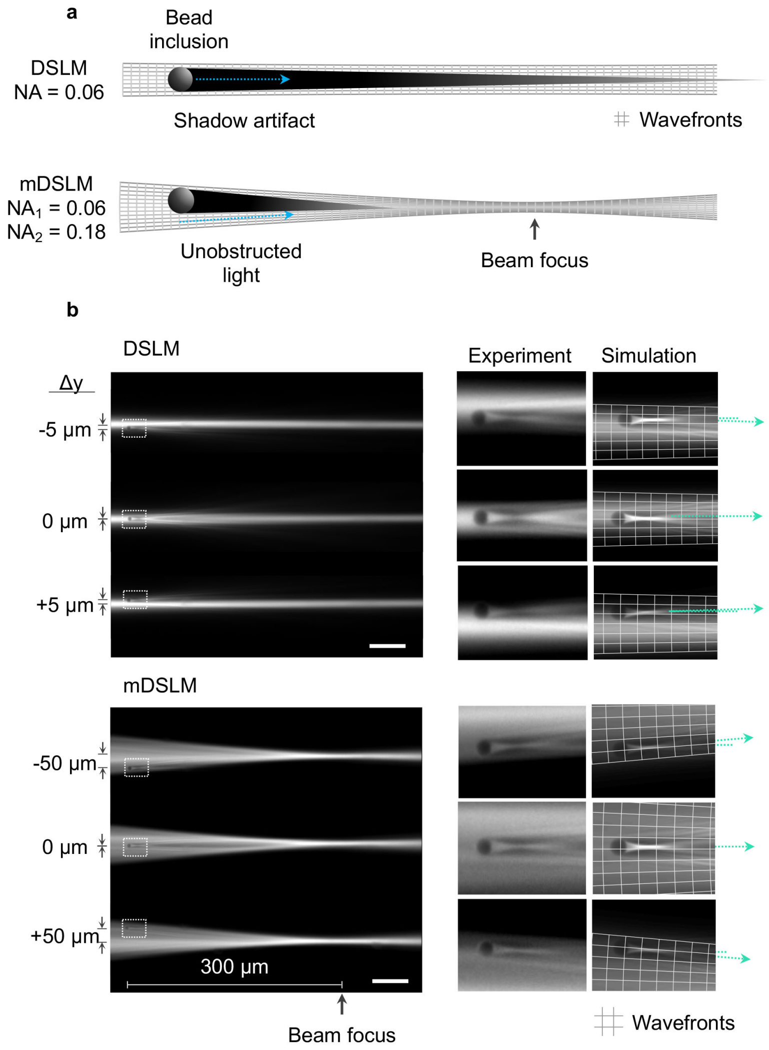

Supplementary Figure 11. Multidirectional illumination. (a) In comparison to conventional DSLM systems which utilize circular Gaussian beams, our OTLS system uses the mDSLM architecture and an elliptical Gaussian beam for mitigation of shadowing artifacts. **(b)** Simulation and corresponding experimental image of a circular Gaussian beam ($NA_1 = NA_2 = 0.06$) and elliptical Gaussian beam ($NA_1 = 0.06$, $NA_2 = 0.18$) propagating around a large glass sphere (diameter d = 6 μm, n_{sphere} = 1.59) embedded within a fluorescent gel (n_{gel} = 1.46). The sphere is positioned at a depth of 25 μm and both beams are focused at a depth of 300 μm. The scale bar in **(b)** represents 50 μm.

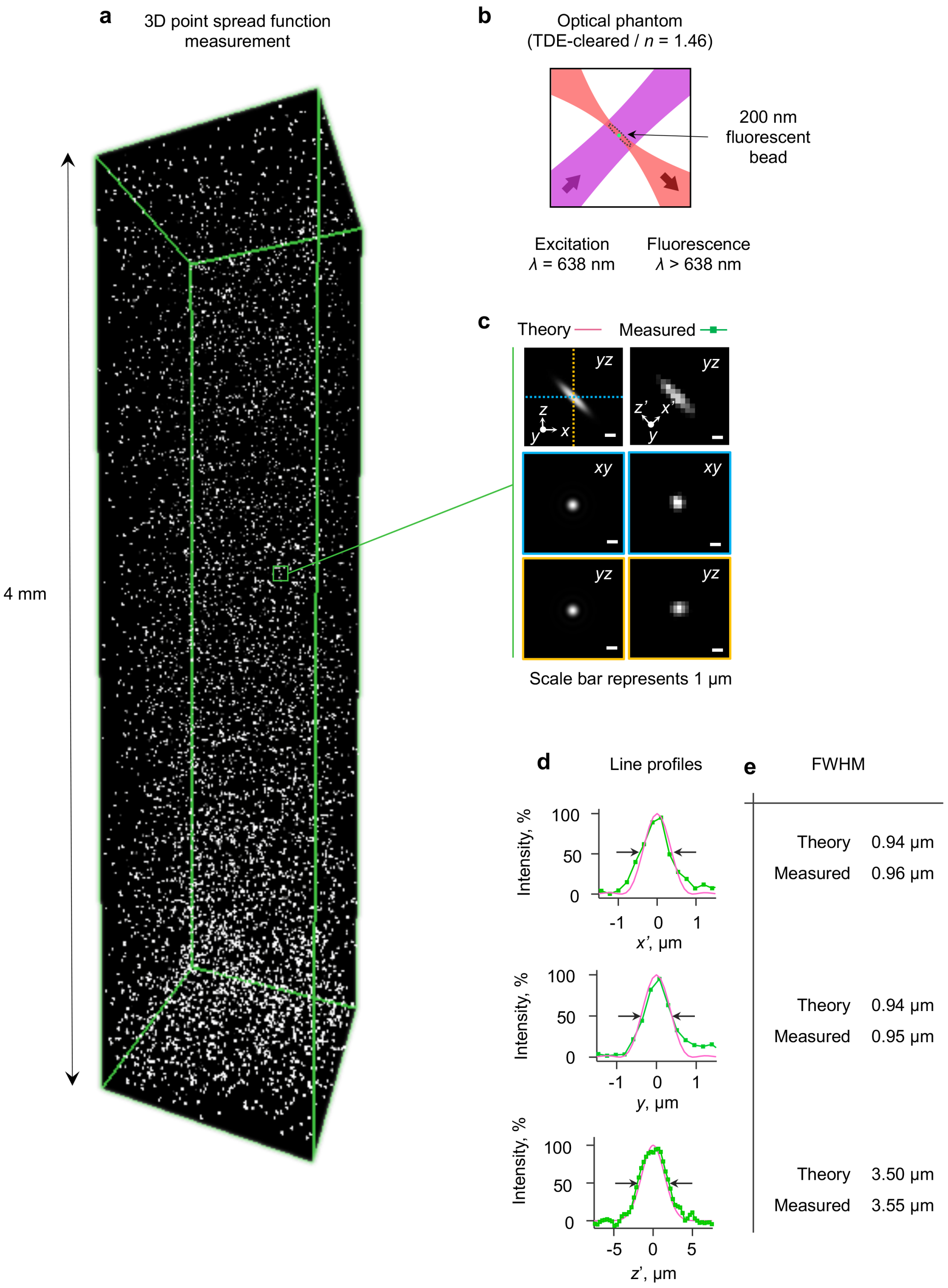

Supplementary Figure 12. PSF measurement. (a) 3D rendering of the point spread function measured in an optical phantom to a depth of 4 mm. **(b)** The optical phantom was comprised with 200 nm fluorescent beads (1:1000 dilution, FSFR002, Bangs Laboratories) in 1% low-melting point agarose. After fabrication, the phantom was TDE-cleared to a refractive index of ~1.46. Line profiles and comparisons of the measured point spread function and theoretical point spread function in **(c)** are shown in **(d)** and **(e)**.

Illumination and collection objective mounting to immersion chamber

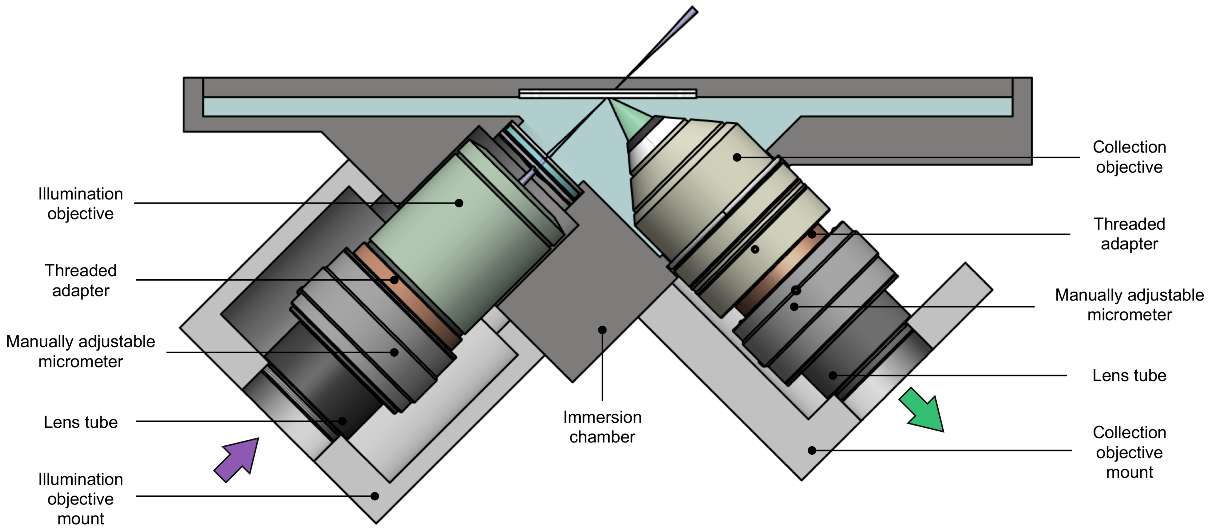

Supplementary Figure 13. Immersion chamber. Both the illumination and collection objectives interface with the immersion chamber using custom fabricated objective mounts. The objectives are each attached to the mounts using threaded adapters, manually adjustable micrometers, and lens tubes. The manually adjustable micrometers enable precise adjustment of the objective distances from the beam focus for alignment purposes. The custom components are available as **Supplementary CAD Files**.

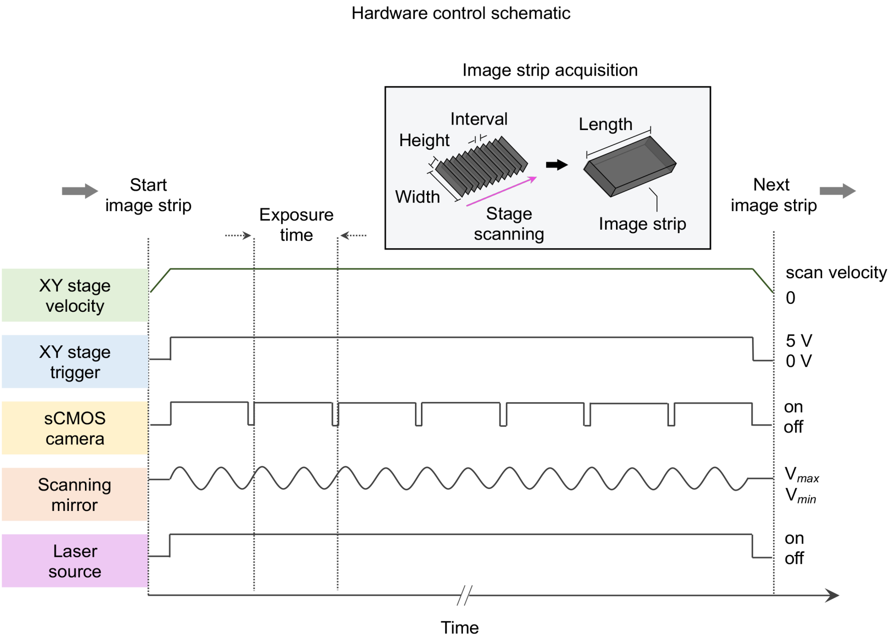

Supplementary Figure 14. Image strip acquisition. Image strips are collected with a combination of stage-scanning and lateral/vertical tiling using a motorized XY stage and Z actuators. The stage-scanning firmware is used to send a TTL trigger signal from the XY stage to the sCMOS camera for reproducible start positioning (<1 µm) of each image strip. The spatial interval between successive frames is set to ~0.32 µm, which given the 800 Hz camera framerate, corresponds to a constant stage velocity of ~0.25 mm/sec. The scanning mirror and laser source are activated at the beginning of an image strip, and deactivated at the end of an image strip.

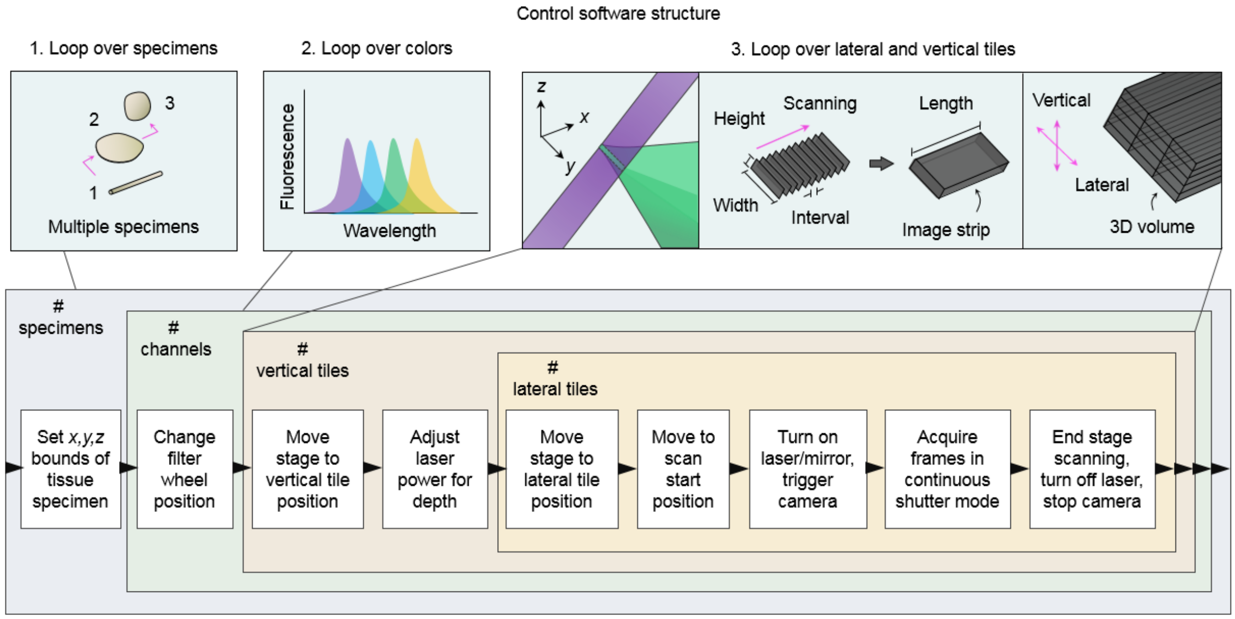

Supplementary Figure 15. Data acquisition scheme. Imaging data is collected by using a series of nested loops. The outermost loop scans over the # of specimens, with user specification of the *x, y, z* bounds of each tissue specimen. The second loop collects the number of user-defined color channels for each specimen. Finally, the innermost loops iterate over the vertical and lateral tiles necessary to cover the entire specimen.

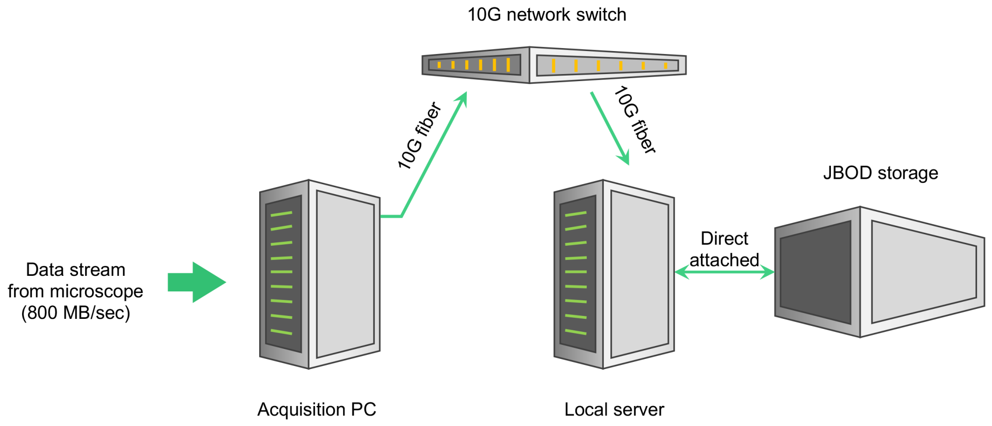

Supplementary Figure 16. Computing hardware. In contrast to conventional microscopes, which typically acquire data at 1 MB/sec onto a single HDD, rely upon CPU processing, and can transfer/store data using 1 Gbit networks to external drives, light-sheet microscopes acquire data at up to 1 GB/sec. This requires specialized hardware, including a RAID array of SSDs, GPU processing, and 10 Gbit transfer to network storage. In our OTLS system, data is acquired and streamed from a low-specification acquisition PC through a 10Gbit network to a high-specification local server with direct-attached JBOD storage. Complete specifications of the system are provided in **Supplementary Table 4**.

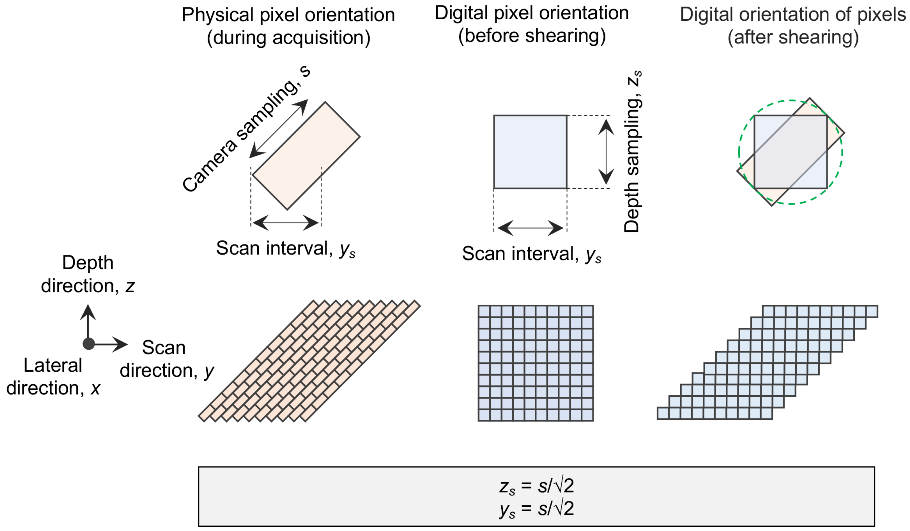

Supplementary Figure 17. Scan interval between successive frames. Raw image frames are collected at an oblique 45-deg angle within specimen. These images are initially oriented at 0 deg in a three-dimensional cube of imaging data. To restore the physical orientation of the image planes, the data must be sheared at the 45 deg angle. Rather than using an affine transformation calculation, we employ a strategy which shits each row of pixels by an integer pixel offset. For this operation to be valid, the scan interval between successive frames, y_s, must be equal to the in-plane camera sampling pitch, s, divided by $\sqrt{2}$**.**

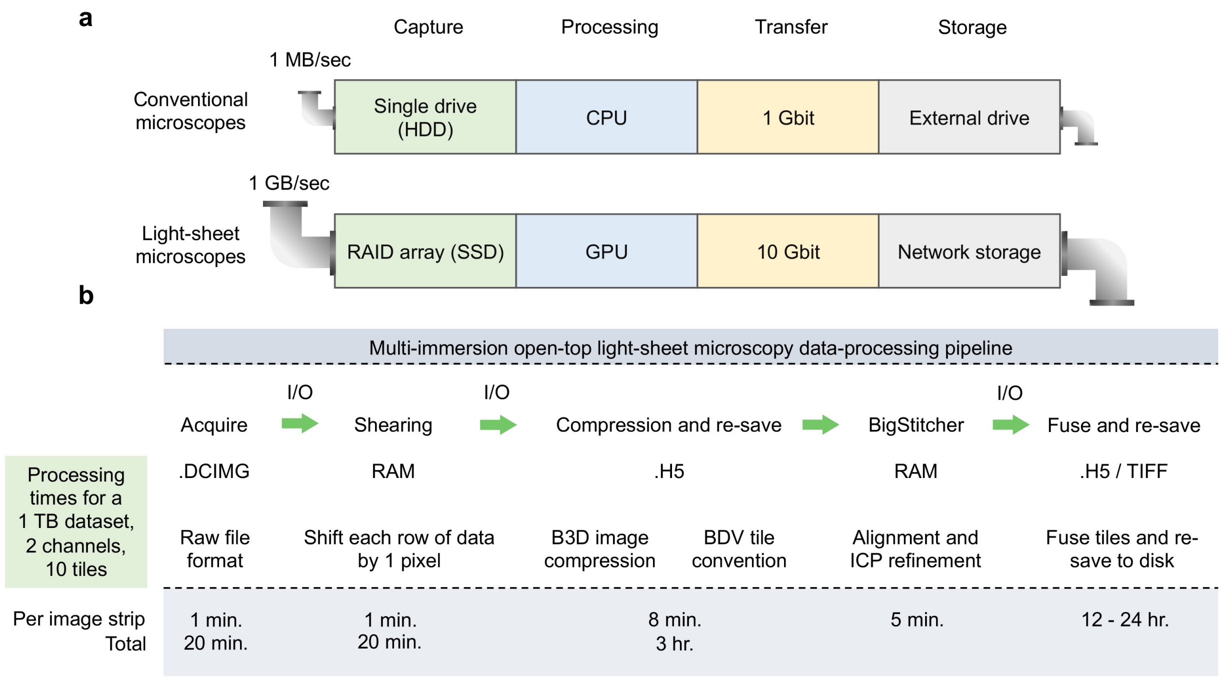

Supplementary Figure 18. Data processing scheme. (a) In contrast to conventional microscopes, which typically acquire data at 1 MB/sec onto a single HDD, rely upon CPU processing, and can transfer/store data using 1 Gbit networks to external drives, light-sheet microscopes acquire data at up to 1 GB/sec. This requires specialized hardware, including a RAID array of SSDs, GPU processing, and 10 Gbit transfer to network storage. (b) For the multi-immersion OTLS system, each image strip is stored in a single DCIMG file. These DCIMG files are read into RAM by a DLL compiled using the Hamamatsu DCIMG software development kit (SDK) and first sheared at 45 deg The data is then written from RAM to disk using the Hierarchical Data Format (HDF5) with the metadata and XML file structured for subsequent analysis using BigStitcher [31]. A custom HDF5 compression filter (B3D) is used with default parameters to provide ~10× compression which is within the noise-limit of the sCMOS camera [32]. This pre-processing routine is applied to all DCIMG files, ultimately resulting in a single HDF5/XML file for BigStitcher. The alignment of all image strips is performed in BigStitcher, and finally fused to disk in either TIFF or HDF5 file formats. The resulting TIFF and HDF5 files are then visualized using open-source and commercial packages, including ImageJ, BigDataViewer, Aivia (DRVision), and Imaris (Bitplane) [33, 34]. Representative processing times for a 1 TB dataset are shown in **(b)**. The processing routines are available as **Supplementary Code Files**.

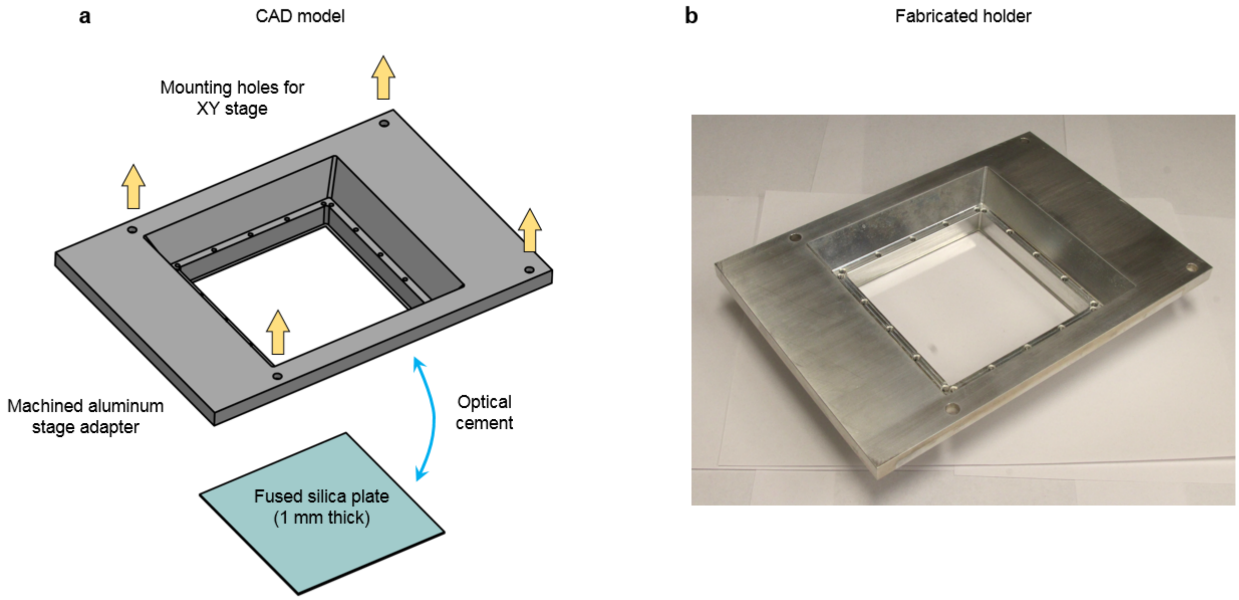

Supplementary Figure 19. TDE-cleared specimen holder. (a) CAD rendering of the specimen holder for TDE-clearing. A 10×10 cm by 1 mm thick fused silica plate is optically cemented into a custom-machined aluminum adapter which can be mounted to the XY stage. An image of the fabricated holder is shown in **(b)**. Files are available as **Supplementary CAD Files**.

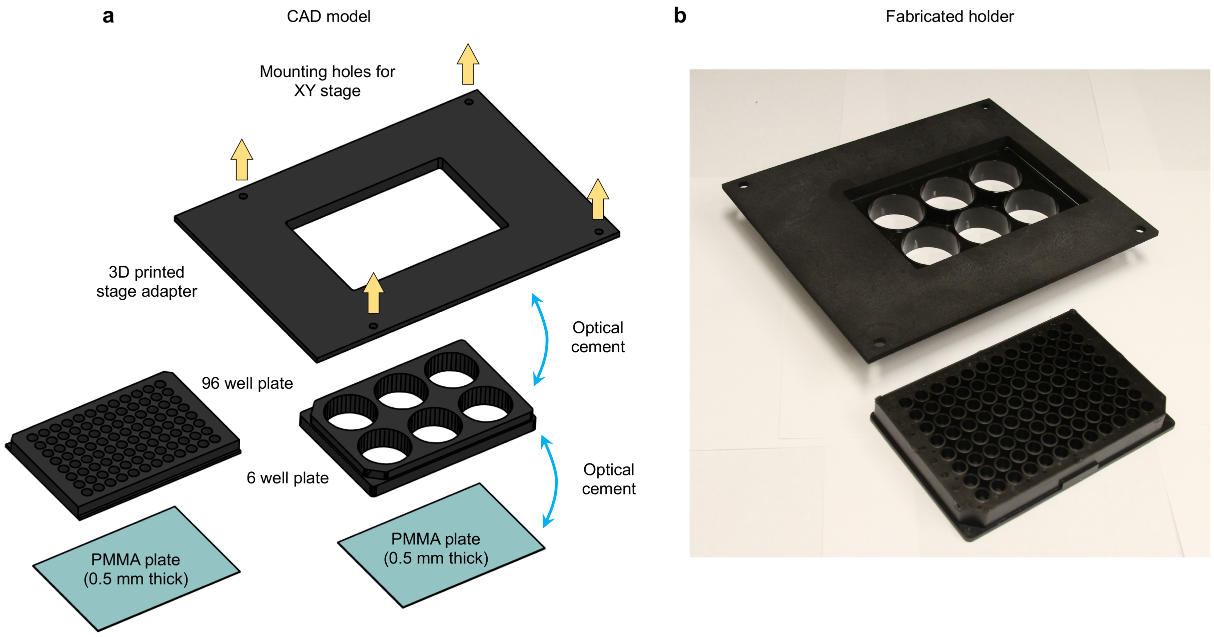

Supplementary Figure 20. Ce3D-cleared specimen holder. (a) CAD rendering of the specimen holder for Ce3D-clearing. The bottom surface of plates (with 96 or 6 wells) are retrofitted with a 0.5 mm thick PMMA plate using optical cement. The plates are then cemented to a 3D printed plastic adapter with holes for mounting to the XY stage. An image of the fabricated holder is shown in **(b)**. Files are available as **Supplementary CAD Files**.

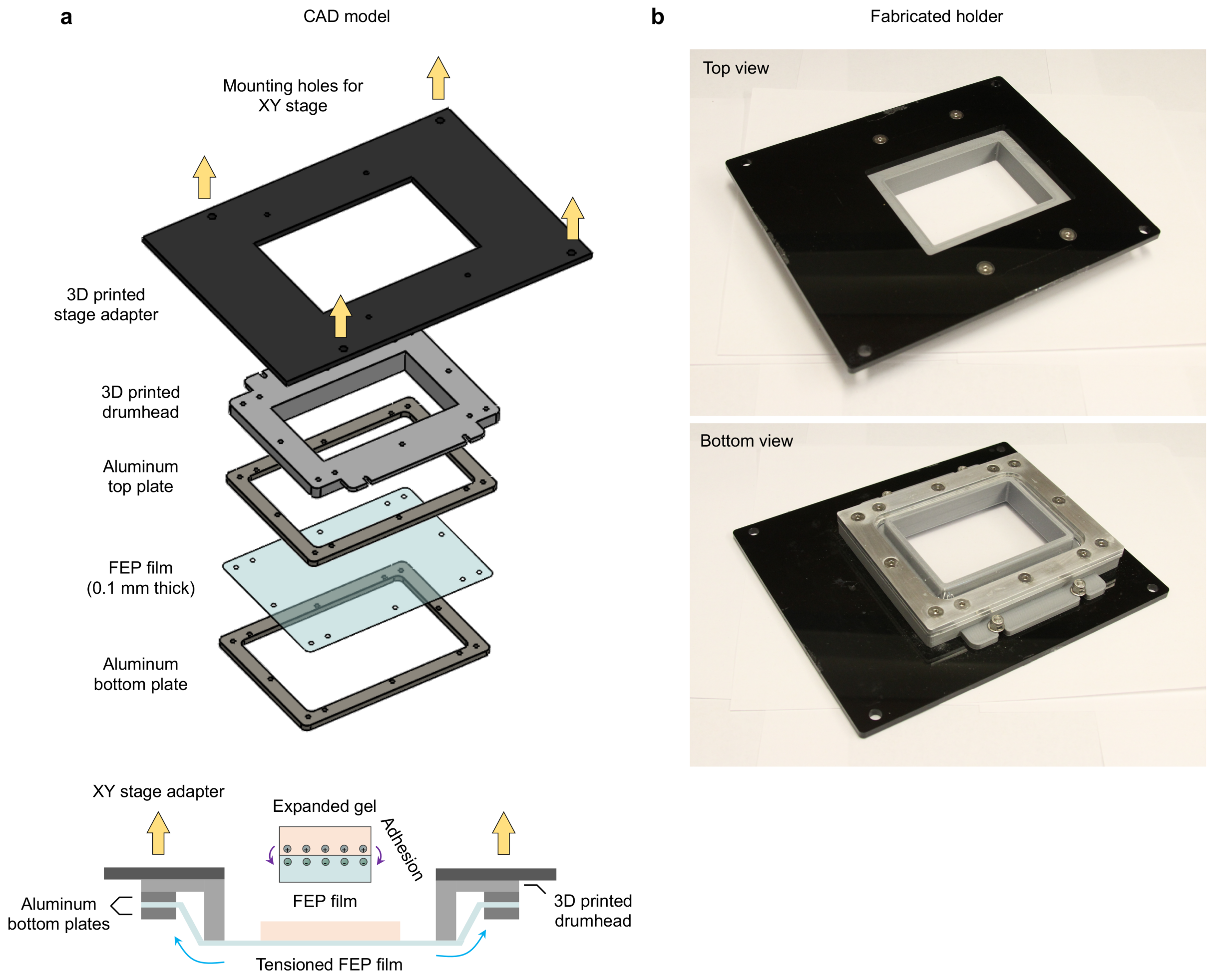

Supplementary Figure 21. ExM specimen holder. (a) CAD rendering of the specimen holder for ExM-clearing. A 0.1 mm thick FEP film is held between two custom machined aluminum plates. The FEP film is then tightened over a 3D printed “drumhead”. The entire drumhead is then attached to a 3D printed mounting plate which attaches to the XY stage. Prior to imaging, the upper surface of the FEP film is treated with poly-lysine to promote adhesion of the expanded gel to the FEP film and prevent movement of the gel during long imaging sessions. Top and bottom images of the fabricated holder is shown in **(b)**. Files are available as **Supplementary CAD Files**.

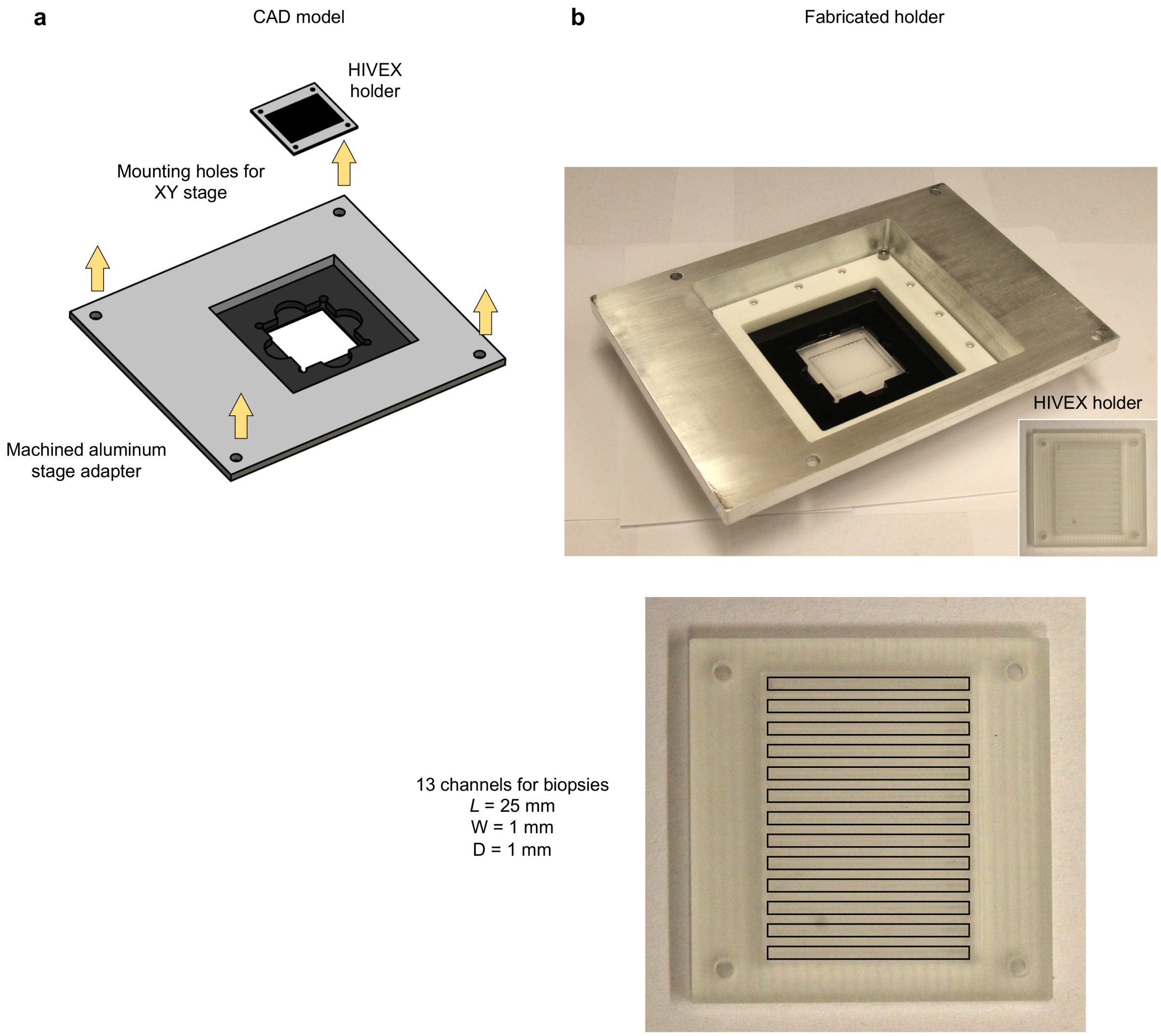

Supplementary Figure 22. ECi-cleared specimen holder. (a) CAD rendering of the specimen holder for ECi-clearing. A custom machined HIVEX holder with 13 channels for human biopsies is placed in a custom machined aluminum adapter for mounting to the XY stage. An image of the fabricated holder is shown in **(b)**. Files are available as **Supplementary CAD Files**.

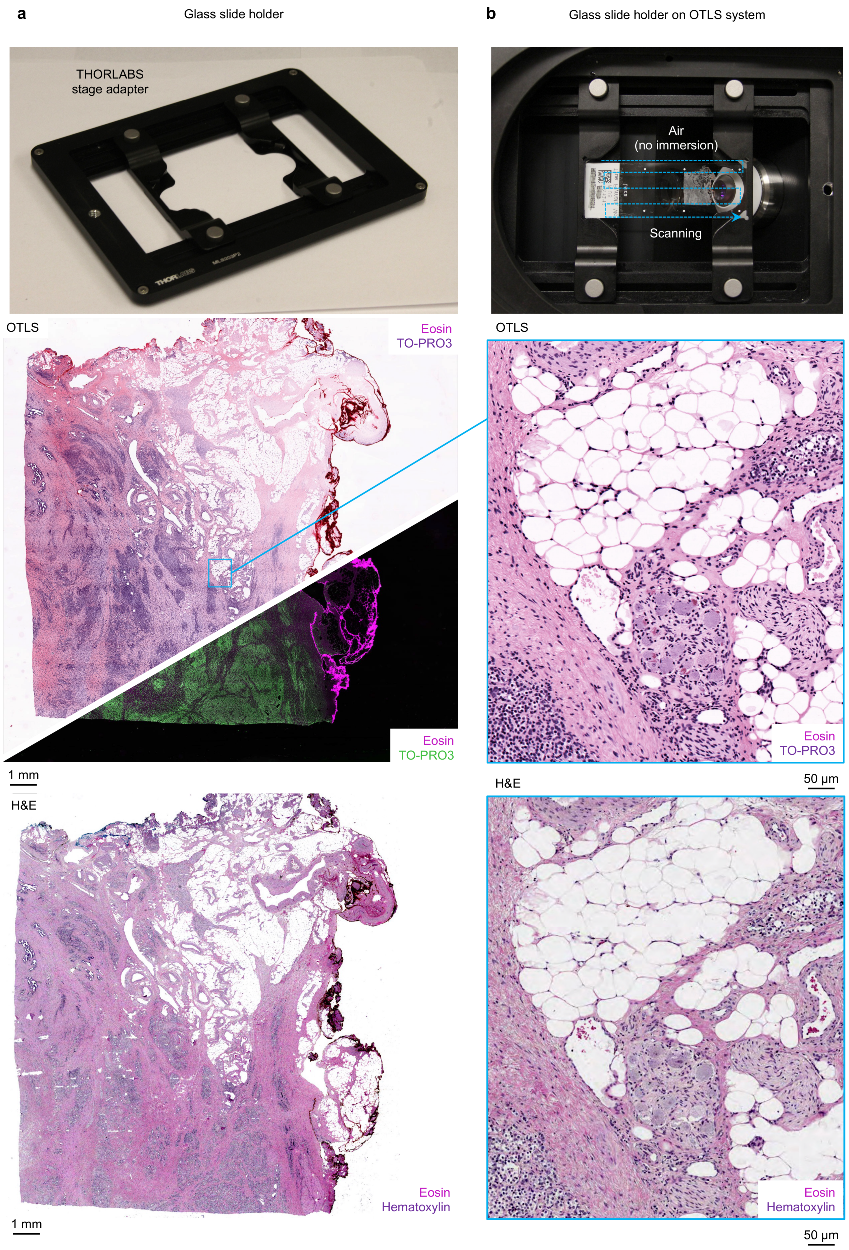

Supplementary Figure 23. Glass slide holder and whole slide imaging. (a) Sage adapter for holding glass slides. **(b)** Representative image of a fluorescently labeled (TO-PRO3 and Eosin) slide-mounted histology section on the OTLS system. Glass slides may be imaged in air (i.e. no immersion) since the tissue sections are thin and optical aberrations only accumulate at larger depths. A whole slide fluorescence image of the slide in **(b)** is shown with pseudo-H&E false coloring (top left) as well as a dark-field false-coloring palette (bottom right) that is more typical for fluorescence microscopy. A high magnification zoom-in is also shown.

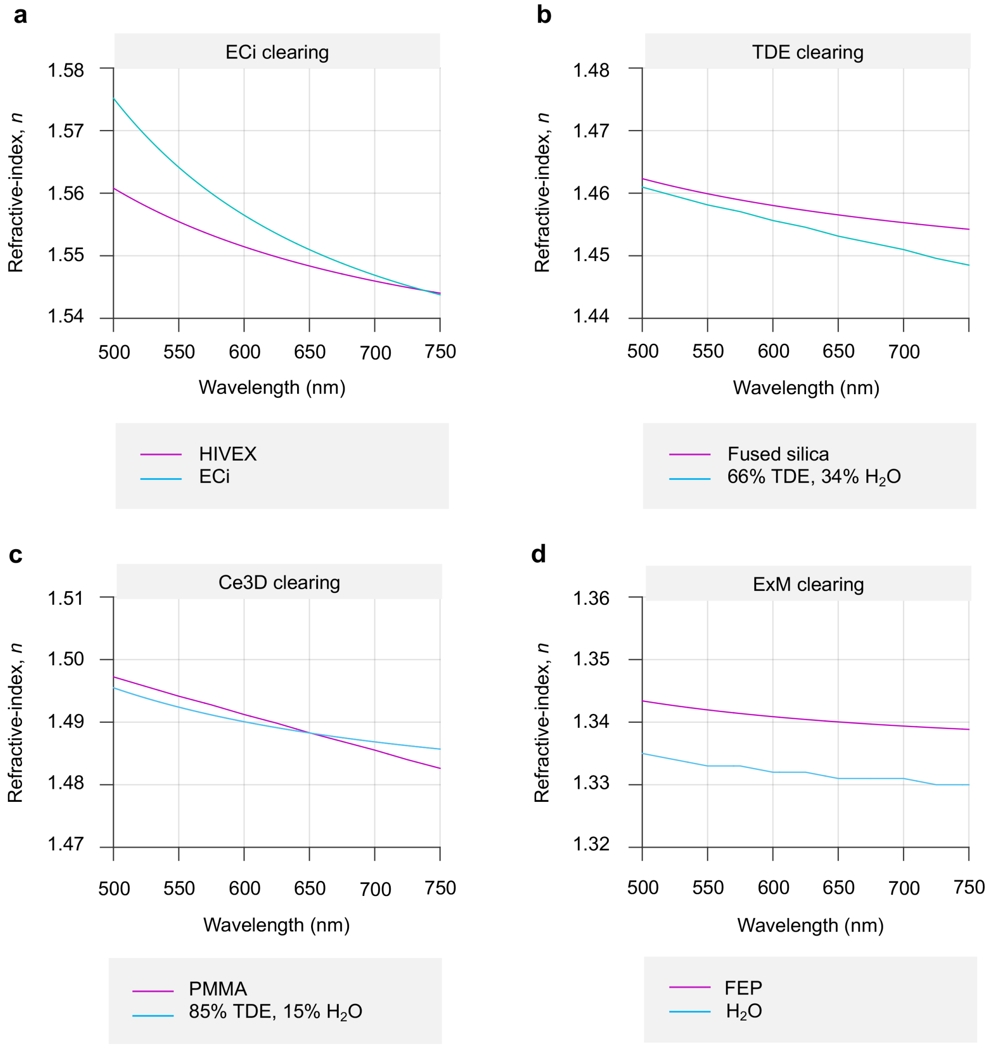

Supplementary Figure 24. Dispersion curves for specimen holder and clearing reagent combinations. (a-d) The dispersion curves for ECi clearing (ECi / HIVEX), TDE clearing (66% TDE, 34% H_2O / Fused silica), Ce3D clearing (85% TDE, 15% H_2O / PMMA), and ExM clearing (H_2O / FEP) are shown. Data for materials and reagents were obtained from [20, 35, 36]. Data for the HIVEX (Conant Optical) and FEP (DuPont) materials were obtained from the manufacturers. However, the dispersion of many clearing reagents, particularly multi-compound aqueous-based solutions, are unknown (see the **Supplementary Discussion**).

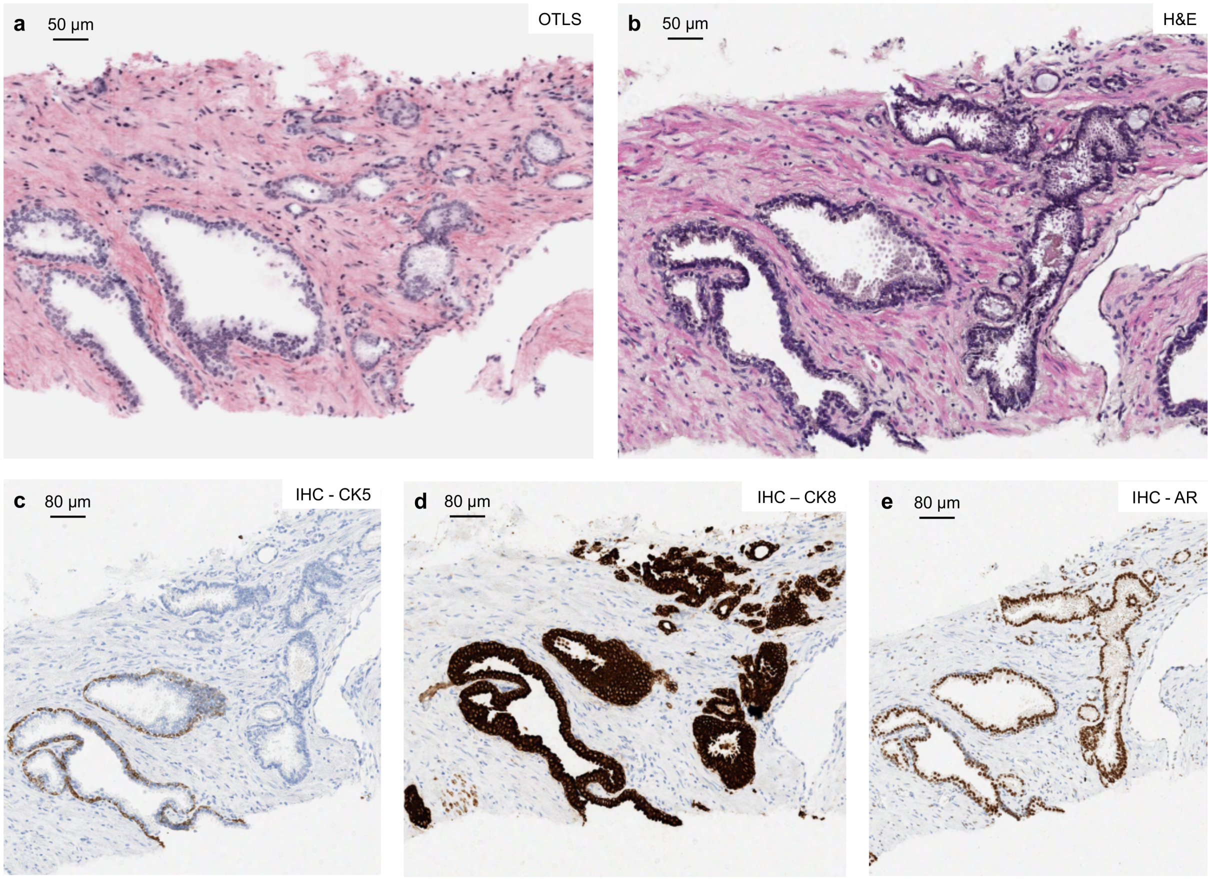

Supplementary Figure 25. Compatibility of ECi-clearing with downstream histology of human prostate tissue. (a) 2D OTLS image of an intact human prostate biopsy. **(b-e)** Corresponding H&E and IHC of the same specimen and region of interest containing a large vascular channel, benign glands, and well-formed carcinoma glands. Nuclear (AR) and cytoplasmic (CK8, CK5) stains are shown. AR stains the nucleus of all carcinoma and benign glands, CK8 stains the cytoplasm of all carcinoma glands and the luminal epithelium of the benign glands, and CK5 stains the basal cells of the benign glands, but is absent in the carcinoma glands.

Supplementary Tables

Supplementary Table 1. Imaging datasets

Dataset Name	Sampling (μm/px)	Dimensions (px)	Size (mm)	Fluorescent Markers	Laser (mW)	Attenuation (mm^{-1})	Time (min)	Size (GB)	B3D (GB)
**TDE clearing (*n* = 1.46)**									
Brain Slice (1)	0.332 (X)<br>0.469 (Z)<br>0.332 (Z)	14912<br>17920<br>624	4.9 (X)<br>8.7 (Y)<br>0.2 (Z)	GFP (488)	6 mW	1.0	13	172	24
Brain Slice (2)	0.332 (X)<br>0.469 (Z)<br>0.332 (Z)	14912<br>17920<br>624	4.9 (X)<br>8.7 (Y)<br>0.2 (Z)	GFP (488)	6 mW	1.0	13	172	25
Brain Slice (3)	0.332 (X)<br>0.469 (Z)<br>0.332 (Z)	14912<br>17920<br>624	4.9 (X)<br>8.7 (Y)<br>0.2 (Z)	GFP (488)	6 mW	1.0	13	172	22
Brain Slice (4)	0.332 (X)<br>0.469 (Z)<br>0.332 (Z)	14912<br>17920<br>624	4.9 (X)<br>8.7 (Y)<br>0.2 (Z)	GFP (488)	6 mW	1.0	13	172	22
Brain Slice (5)	0.332 (X)<br>0.469 (Z)<br>0.332 (Z)	14912<br>17920<br>624	4.9 (X)<br>8.7 (Y)<br>0.2 (Z)	GFP (488)	6 mW	1.0	13	172	22
Brain Slice (6)	0.332 (X)<br>0.469 (Z)<br>0.332 (Z)	14912<br>17920<br>624	4.9 (X)<br>8.7 (Y)<br>0.2 (Z)	GFP (488)	6 mW	1.0	13	172	23
Brain Slice (7)	0.332 (X)<br>0.469 (Z)<br>0.332 (Z)	14912<br>17920<br>624	4.9 (X)<br>8.7 (Y)<br>0.2 (Z)	GFP (488)	6 mW	1.0	13	172	26
Brain Slice (8)	0.332 (X)<br>0.469 (Z)<br>0.332 (Z)	14912<br>17920<br>624	4.9 (X)<br>8.7 (Y)<br>0.2 (Z)	GFP (488)	6 mW	1.0	13	172	22
**Ce3D clearing (*n* = 1.49)**									
Mouse Lung	0.326 (X)<br>0.460 (Y)<br>0.326 (Z)	12261 (X)<br>24030 (Y)<br>2686 (Z)	4.1 (X)<br>11.3 (Y)<br>0.9 (Z)	F-actin (561)<br>EpCAM (638)	10 mW<br>1 mW	1.0<br>1.0	118	3165	473
Mouse Heart	0.652 (X)<br>0.920 (Y)<br>0.652 (Z)<br>(2x binning)	6617 (X)<br>6744 (Y)<br>5970 (Z)	4.4 (X)<br>6.4 (Y)<br>4.0 (Z)	AF (488)<br>DRAQ5 (638)	20 mW<br>2 mW	1.0<br>1.0	41	532	67
Mouse Prostate	0.326 (X)<br>0.460 (Y)<br>0.326 (Z)	14464 (X)<br>10688 (Y)<br>1472 (Z)	4.8 (X)<br>5.0 (Y)<br>0.5 (Z)	CK8 (638)	8 mW	1.0	18	228	32
Lymph Node (1)	0.326 (X)<br>0.460 (Y)<br>0.326 (Z)	5248 (X)<br>2880 (Y)<br>1472 (Z)	1.8 (X)<br>1.4 (Y)<br>0.5 (Z)	CD3 (405)<br>CD11 (488)<br>F-actin (561)<br>B220 (638)	2 mW<br>8 mW<br>1 mW<br>20 mW	1.0<br>1.0<br>1.0<br>1.0	9	89	14
Lymph Node (2)	0.326 (X)<br>0.460 (Y)<br>0.326 (Z)	6272 (X)<br>2880 (Y)<br>1472 (Z)	2.1 (X)<br>1.4 (Y)<br>0.5 (Z)	CD3 (405)<br>CD11 (488)<br>F-actin (561)<br>B220 (638)	2 mW<br>8 mW<br>1 mW<br>20 mW	1.0<br>1.0<br>1.0<br>1.0	10	106	15

Lymph Node (3)	0.326 (X)<br>0.460 (Y)<br>0.326 (Z)	6272 (X)<br>2944 (Y)<br>1472 (Z)	2.1 (X)<br>1.4 (Y)<br>0.5 (Z)	CD3 (405)<br>CD11 (488)<br>F-actin (561)<br>B220 (638)	2 mW<br>8 mW<br>1 mW<br>20 mW	1.0<br>1.0<br>1.0<br>1.0	10	110	16.7
Lymph Node (4)	0.326 (X)<br>0.460 (Y)<br>0.326 (Z)	5248 (X)<br>2880 (Y)<br>1984 (Z)	1.8 (X)<br>1.4 (Y)<br>0.7 (Z)	CD3 (405)<br>CD11 (488)<br>F-actin (561)<br>B220 (638)	2 mW<br>8 mW<br>1 mW<br>20 mW	1.0<br>1.0<br>1.0<br>1.0	12	120	18
Lymph Node (5)	0.326 (X)<br>0.460 (Y)<br>0.326 (Z)	6656 (X)<br>2880 (Y)<br>2432 (Z)	2.2 (X)<br>1.5 (Y)<br>0.8 (Z)	CD3 (405)<br>CD11 (488)<br>F-actin (561)<br>B220 (638)	2 mW<br>8 mW<br>1 mW<br>20 mW	1.0<br>1.0<br>1.0<br>1.0	17	186	24
Lymph Node (6)	0.326 (X)<br>0.460 (Y)<br>0.326 (Z)	6144 (X)<br>2944 (Y)<br>1984 (Z)	2.1 (X)<br>1.5 (Y)<br>0.7 (Z)	CD3 (405)<br>CD11 (488)<br>F-actin (561)<br>B220 (638)	2 mW<br>8 mW<br>1 mW<br>20 mW	1.0<br>1.0<br>1.0<br>1.0	14	144	19
Lymph Node (7)	0.326 (X)<br>0.460 (Y)<br>0.326 (Z)	4352 (X)<br>2880 (Y)<br>1984 (Z)	1.5 (X)<br>1.5 (Y)<br>0.7 (Z)	CD3 (405)<br>CD11 (488)<br>F-actin (561)<br>B220 (638)	2 mW<br>8 mW<br>1 mW<br>20 mW	1.0<br>1.0<br>1.0<br>1.0	11	99	14
Lymph Node (8)	0.326 (X)<br>0.460 (Y)<br>0.326 (Z)	4864 (X)<br>2880 (Y)<br>1920 (Z)	1.6 (X)<br>1.5 (Y)<br>0.7 (Z)	CD3 (405)<br>CD11 (488)<br>F-actin (561)<br>B220 (638)	2 mW<br>8 mW<br>1 mW<br>20 mW	1.0<br>1.0<br>1.0<br>1.0	11	70	9
**ECi clearing (*n* = 1.56)**									
Prostate Biopsy (1)	0.311 (X)<br>0.439 (Y)<br>0.311 (Z)	56872 (X)<br>2064 (Y)<br>2049 (Z)	18.2 (X)<br>0.9 (Y)<br>0.6 (Z)	Eosin (561)<br>TO-PRO3 (638)	1 mW<br>1 mW	0.7<br>0.7	25	962	122
Prostate Biopsy (2)	0.311 (X)<br>0.439 (Y)<br>0.311 (Z)	50155 (X)<br>2063 (Y)<br>2049 (Z)	16.1 (X)<br>0.9 (Y)<br>0.6 (Z)	Eosin (561)<br>TO-PRO3 (638)	1 mW<br>1 mW	0.7<br>0.7	23	848	108
Prostate Biopsy (3)	0.311 (X)<br>0.439 (Y)<br>0.311 (Z)	39564 (X)<br>2074 (Y)<br>2048 (Z)	12.7 (X)<br>0.9 (Y)<br>0.6 (Z)	Eosin (561)<br>TO-PRO3 (638)	1 mW<br>1 mW	0.7<br>0.7	18	669	90
Prostate Biopsy (4)	0.311 (X)<br>0.439 (Y)<br>0.311 (Z)	47364 (X)<br>2066 (Y)<br>2039 (Z)	15.2 (X)<br>0.9 (Y)<br>0.6 (Z)	Eosin (561)<br>TO-PRO3 (638)	1 mW<br>1 mW	0.7<br>0.7	22	801	121
Prostate Biopsy (5)	0.311 (X)<br>0.439 (Y)<br>0.311 (Z)	53271 (X)<br>2061 (Y)<br>2047 (Z)	17.1 (X)<br>0.9 (Y)<br>0.6 (Z)	Eosin (561)<br>TO-PRO3 (638)	1 mW<br>1 mW	0.7<br>0.7	24	901	130
Prostate Biopsy (6)	0.311 (X)<br>0.439 (Y)<br>0.311 (Z)	44860 (X)<br>2066 (Y)<br>2040 (Z)	14.4 (X)<br>0.9 (Y)<br>0.6 (Z)	Eosin (561)<br>TO-PRO3 (638)	1 mW<br>1 mW	0.7<br>0.7	21	758	104
Prostate Biopsy (7)	0.311 (X)<br>0.439 (Y)<br>0.311 (Z)	49844 (X)<br>2062 (Y)<br>2040 (Z)	16.0 (X)<br>0.9 (Y)<br>0.6 (Z)	Eosin (561)<br>TO-PRO3 (638)	1 mW<br>1 mW	0.7<br>0.7	23	843	106
Prostate Biopsy (8)	0.311 (X)<br>0.439 (Y)<br>0.311 (Z)	51714 (X)<br>2066 (Y)<br>2048 (Z)	16.6 (X)<br>0.9 (Y)<br>0.6 (Z)	Eosin (561)<br>TO-PRO3 (638)	1 mW<br>1 mW	0.7<br>0.7	24	874	110
Prostate Biopsy (9)	0.311 (X)<br>0.439 (Y)<br>0.311 (Z)	49221 (X)<br>2069 (Y)<br>2040 (Z)	15.8 (X)<br>0.9 (Y)<br>0.6 (Z)	Eosin (561)<br>TO-PRO3 (638)	1 mW<br>1 mW	0.7<br>0.7	22	832	124

Prostate Biopsy (10)	0.311 (X)<br>0.439 (Y)<br>0.311 (Z)	56075 (X)<br>2062 (Y)<br>2042 (Z)	18.0 (X)<br>0.9 (Y)<br>0.6 (Z)	Eosin (561)<br>TO-PRO3 (638)	1 mW<br>1 mW	0.7<br>0.7	25	948	119
Prostate Biopsy (11)	0.311 (X)<br>0.439 (Y)<br>0.311 (Z)	45483 (X)<br>2066 (Y)<br>2046(Z)	14.6 (X)<br>0.9 (Y)<br>0.6 (Z)	Eosin (561)<br>TO-PRO3 (638)	1 mW<br>1 mW	0.7<br>0.7	22	769	99
Prostate Biopsy (12)	0.311 (X)<br>0.439 (Y)<br>0.311 (Z)	47975 (X)<br>2060 (Y)<br>2040 (Z)	15.4 (X)<br>0.9 (Y)<br>0.6 (Z)	CK8 (488)<br>TO-PRO3 (638)	10 mW<br>1 mW	0.7<br>0.7	23	811	102
**ExM (*n* = 1.33)**									
Mouse Kidney (1)	1.46 (X)<br>2.06 (Y)<br>1.46 (Z)<br>(4x binning)	21192 (X)<br>9859 (Y)<br>728 (Z)	32.0 (Y)<br>21.0 (X)<br>1.1 (Z)	DAPI (405)<br>WGA-lectin (488)<br>Podxl (561)<br>Coll IV (638)	1 mW<br>30 mW<br>50 mW<br>50 mW	2.0<br>2.0<br>2.0<br>2.0	64	608	84
Mouse Kidney (2)	0.365 (X)<br>0.515 (Y)<br>0.365 (Z)	4884 (X)<br>4018 (Y)<br>2918 (Z)	1.8 (X)<br>2.1 (Y)<br>1.1 (Z)	DAPI (405)<br>WGA-lectin (488)<br>Podxl (561)<br>Coll IV (638)	1 mW<br>30 mW<br>50 mW<br>50 mW	2.0<br>2.0<br>2.0<br>2.0	26	229	30

Supplementary Table 2. Immersion medium specifications

Refractive index	Reagents	Cost to fill chamber (300 mL)
n = 1.33	100% DI water	N/A
n = 1.46	66% TDE + 34% DI water	~ 17 USD
n = 1.49	85% TDE + 15% DI water	~20 USD
n = 1.56	100% ECi	~ 60 USD

Due to the design of the OTLS system, the specimen does not contaminate the immersion medium during imaging. Therefore, the immersion medium can be reused for multiple imaging experiments.

Supplementary Table 3. System components list

Part Number	Vendor	Notes	Quantity
**Immersion Chamber**			
IMMERSION CHAMBER	Hilltop Technologies	CAD file provided	1
ILLUMINATION OBJECTIVE MOUNT	Hilltop Technologies	CAD file provided	1
COLLECTION OBJECTIVE MOUNT	Hilltop Technologies	CAD file provided	1
P8	Thorlabs	Immersion chamber supports	4
PB4	Thorlabs	Support post pedestals	4
**Illumination Objective**			
XLFLUOR 340/4X	Olympus	Illumination objective	1
LA4725-A	Thorlabs	Illumination SIL	1
0.945 X 0.030 75 FLUORCARBON	Apple Rubber	SIL gasket	1
SM1RR	Thorlabs	Retaining ring for holding illumination SIL	1
**Collection Objective**			
MULTI-IMMERSION OBJECTIVE	ASI / Special Optics	Collection objective NA = 0.40 @ n = 1.45 (range n = 1.33 – 1.56)	1
1.461 X 0.063 70 BUNA-N	Apple Rubber	Collection objective gasket	1
**Illumination Optics**			
AC127-019-A-ML	Thorlabs	Collimating lens f = 19 mm	1
ACY254-50-A	Thorlabs	Cylindrical lens f = 50 mm	1
ACY254-150-A	Thorlabs	Cylindrical lens f = 150 mm	1
AC254-75-A-ML	Thorlabs	Relay lenses f = 75 m	2
CLS-SL	Thorlabs	Scan lens f = 70 mm	1
TTL200-A	Thorlabs	Tube lens f = 200 mm	1
**Collection Optics**			
TTL165-A	Thorlabs	Tube lens f = 165 mm	1
SM2F	Thorlabs	Adjustable collimation adapter for tube lens	1
**Imaging Camera**			
ORCA-FLASH4.0 V2	Hamamatsu	sCMOS camera	1
**Filter Wheel**			
FW102C	Thorlabs	Motorized filter wheel	1
FF02-447/60-25	Semrock	405 bandpass filter	1
FF03-525/50-25	Semrock	488 bandpass filter	1
FF01-618/50-25	Semrock	561 bandpass filter	1
FF01-721/65-25	Semrock	638 bandpass filter	1
**Scanning Stage Components**			
MS-2000 XY STAGE	ASI	Motorized XY stage, modified top-plate for open-top mounting	1
LS-50 Z TRANSLATORS	ASI	Motorized Z axis translators	2
P4	Thorlabs	Breadboard supports	4
PB4	Thorlabs	Support post pedestals	4

TR2.5	Thorlabs	LS50 raisers	8
PH2.5	Thorlabs	LS50 raisers	8
**Customized Specimen Holders**			
FLEXVAT HOLDER AND STAGE ADAPTER	Flexvat.com	For holding FEP film and expanded specimens (*n* = 1.33) CAD file provided	1
FLAT PLATE STAGE ADAPTER	Hilltop Technologies	For holding fused silica plate (*n* = 1.46) CAD file provided	1
WELL PLATE STAGE ADAPTER	Hilltop Technologies	For holding well plate with PMMA bottom (*n* = 1.49) CAD file provided	1
BIOPSY STAGE ADAPTER	Hilltop Technologies	For holding HIVEX biopsy holder (*n* = 1.56) CAD file provided	1
HIVEX HOLDER	In house	For holding biopsies (*n* = 1.56) CAD file provided	1
**Laser Package**			
90420	Cobolt	Skyra fiber-coupled 405, 488, 561, 638 lasers	1
12422	Cobolt	Heatsink	1
PM-S405-XP-CUSTOM	Thorlabs	Custom FC/APC FC/PC S405-XP fiber	1
**Scanning Mirror**			
6210H	Cambridge Technologies	Galvanometer mirror	1
6210H Mount	Cambridge Technologies	Mirror mount	1
NI-6115	National Instruments	Waveform generator	1
**Optomechanical Assembly Components**			
SM1A61	Thorlabs	-	1
SM1ZM	Thorlabs	-	2
SM1A24	Thorlabs	-	1
SM1L05	Thorlabs	-	1
SM1Z	Thorlabs	-	1
CXY1	Thorlabs	-	1
LCP02	Thorlabs	-	8
CRM1-P	Thorlabs	-	2
CPB1	Thorlabs	-	5
LCP01	Thorlabs	-	9
CP02	Thorlabs	-	1
KCB2EC	Thorlabs	-	4
PFE20-P01	Thorlabs	-	5
ER3	Thorlabs	-	5
ER12	Thorlabs	-	8
ER10	Thorlabs	-	4
ER4	Thorlabs	-	16
ER2	Thorlabs	-	8
SM1FC	Thorlabs	-	1
SM1A6	Thorlabs	-	1

C6W	Thorlabs	-	1
LB4C	Thorlabs	-	1
SM2F	Thorlabs	-	1
SM2A55	Thorlabs	-	1
TR075	Thorlabs	-	3
PH1	Thorlabs	-	3
MF469-35	Thorlabs	-	1
PF175	Thorlabs	-	6
AP45	Thorlabs	-	8
SM1A12	Thorlabs	-	1
M32M34S	Thorlabs	-	1
LCPB1	Thorlabs	-	4
SM2A20	Thorlabs	-	1
KCB1E	Thorlabs	-	1
SMA2A55	Thorlabs	-	1
MB1236	Thorlabs	-	2
TR2	Thorlabs	-	1
PH1.5	Thorlabs	-	1

Supplementary Table 4. Computer hardware specifications

Part Number	Vendor	Notes	Quantity
**Acquisition Computer**			
Precision Tower 5810	Dell	1x CPU<br>2 PCI-E 3.0 x16 (double-width) slots,<br>1 PCI-E 3.0 x8 (single-width) slots,<br>1 PCI-E 2.0 x4 slot<br>1 PCI-E 2.0 x1 slot<br>4 3.5" drive bays<br>USB3.0 (1 front port)<br>USB2.0 (3 front ports)<br>USB3.0 (3 rear ports)<br>USB2.0 (3 internal ports)	1
SSD 2.00 TB 960 PRO Series	Samsung	2.5" SATA 6.0Gb/s Solid State Drive (RAID0)	4
MegaRAID 9361-8i	LSI	SAS 12Gb/s PCIe 3.0 8-Port Controller with 1GB Cache (1x internal RAID0 arrays)	1
GPU Quadro K620	NVIDIA	12 GB GDDR5X (administrator account)	1
10-Gigabit Ethernet Adapter MCX311A	Mellanox	ConnectX-3 EN MCX311A (1x SFP+)	1
RAM KVR21E15D8/16	Kingston	16 GB ECC Registered DDR4 2133 PC4 1700	2
FireBird 1xCLD-2PE8	Active Silicon	PCIe 3.0 x8 sCMOS camera frame grabber	1
Windows 7	Microsoft	64-bit	1
**Computing Server**			
SuperWorkstation 7049GP-TRT	SuperMicro	2x CPUs<br>4 PCI-E 3.0 x16 (double-width) slots,<br>2 PCI-E 3.0 x16 (single-width) slots,<br>1 PCI-E 3.0 x4 (in x8) slot<br>8 Hot-swap 3.5" drive bays<br>Up to 2TB ECC 3DS LRDIMM, up to DDR4-2666MHz; 16 DIMM slots<br>Dual socket P (LGA 3647) supports Intel® Xeon® Scalable Processors	1
CPU Xeon Gold 6134	Intel	8-core 3.20GHz 24.75MB Cache (130W)	2
NVMe SSD 960 PRO M.2	Samsung	512GB PCIe 3.0 x4 NVMe (1 Cache, 1 OS)	2
SSD 1.92TB 5200 ECO Series	Micron	2.5" SATA 6.0Gb/s Solid State Drive (RAID0)	4
SSD 2.00 TB 960 PRO Series	Samsung	2.5" SATA 6.0Gb/s Solid State Drive (RAID0)	4
MegaRAID 9361-8i	LSI	SAS 12Gb/s PCIe 3.0 8-Port Controller with 1GB Cache (2x internal RAID0 arrays)	1
MegaRAID 9380-8e	LSI	SAS 12Gb/s PCIe 3.0 8-Port Controller with 1GB Cache (1x external RAID6 array)	1
10-Gigabit Ethernet Adapter MCX311A	Mellanox	ConnectX-3 EN MCX311A (1x SFP+)	1
RAM KTD-PE421LQ/32G	Kingston	32 GB ECC Registered DDR4 2133 PC4 1700	12
GPU TitanXP	NVIDIA	12 GB GDDR5X (administrator account)	1
GPU Quadro P6000	NVIDIA	24 GB GDDR5X (discrete device assignment guest account)	1
OS Windows Server 2016	Microsoft	64-bit	1

Direct-attached Storage			
Chasis STX-3316 3U	Thinkmate	16x Hot-Swap 3.5" SATA/SAS3 12Gb/s SAS Single Expander	1
Exos 7E8 Series (512e)	Seagate	8.0TB SAS 3.0 12.0Gb/s 7200RPM - 3.5" (RAID6 array 96 TB total storage)	16
External SAS Cable	Thinkmate	1-meter 12Gb/s to 12Gb/s SAS - SFF-8644 to SFF-8644	1

Supplementary References

1. Ertürk, A., et al., *Three-dimensional imaging of solvent-cleared organs using 3DISCO.* Nature Protocols, 2012. **7**: p. 1983-1995.
2. Pan, C., et al., *Shrinkage-mediated imaging of entire organs and organisms using uDISCO.* Nature Methods, 2016. **13**: p. 859-867.
3. Renier, N., et al., *iDISCO: a simple, rapid method to immunolabel large tissue samples for volume imaging.* Cell, 2014. **159**(4): p. 896-910.
4. Becker, K., et al., *Chemical Clearing and Dehydration of GFP Expressing Mouse Brains*, in *PLoS One*. 2012.
5. Jing, D., et al., *Tissue clearing of both hard and soft tissue organs with the PEGASOS method.* Cell Research, 2018. **28**(8): p. 803.
6. Klingberg, A., et al., *Fully Automated Evaluation of Total Glomerular Number and Capillary Tuft Size in Nephritic Kidneys Using Lightsheet Microscopy.* Journal of the American Society of Nephrology, 2016. **28**(2): p. 452-459.
7. Hou, B., et al., *Scalable and DiI-compatible optical clearance of the mammalian brain.* Front in Neuroanatomy, 2015. **9**.
8. Li, W., R.N. Germain, and M.Y. Gerner, *Multiplex, quantitative cellular analysis in large tissue volumes with clearing-enhanced 3D microscopy (Ce3D).* Proceedings of the National Academy of Sciences of the United States of America, 2017. **114**(35): p. E7321-e7330.
9. Ke, M.-T., S. Fujimoto, and T. Imai, *SeeDB: a simple and morphology-preserving optical clearing agent for neuronal circuit reconstruction.* Nature Neuroscience, 2013. **16**: p. 1154-1161.
10. Susaki, E.A., et al., *Advanced CUBIC protocols for whole-brain and whole-body clearing and imaging.* Nature Protocols, 2015. **10**: p. 1709-1727.
11. Susaki, E.A., et al., *Whole-brain imaging with single-cell resolution using chemical cocktails and computational analysis.* Cell, 2014. **157**(3): p. 726-39.
12. Costantini, I., et al., *A versatile clearing agent for multi-modal brain imaging.* Scientific Reports, 2015. **5**: p. 9808.
13. Kim, S.Y., et al., *Stochastic electrotransport selectively enhances the transport of highly electromobile molecules.* Proceedings of the National Academy of Sciences of the United States of America, 2015. **112**(46): p. E6274-83.
14. Treweek, J.B., et al., *Whole-body tissue stabilization and selective extractions via tissue-hydrogel hybrids for high-resolution intact circuit mapping and phenotyping.* Nature Protocols, 2015. **10**(11): p. 1860-96.
15. Tomer, R., et al., *Advanced CLARITY for rapid and high-resolution imaging of intact tissues.* Nature Protocols, 2014. **9**(7): p. 1682-97.
16. Chung, K. and K. Deisseroth, *CLARITY for mapping the nervous system.* Nature Methods, 2013. **10**: p. 508-513.
17. Kuwajima, T., et al., *ClearT: a detergent- and solvent-free clearing method for neuronal and non-neuronal tissue.* Development, 2013. **140**(6): p. 1364-8.
18. Hama, H., et al., *Scale: a chemical approach for fluorescence imaging and reconstruction of transparent mouse brain.* Nature Neuroscience, 2011. **14**(11): p. 1481-8.
19. Murray, E., et al., *Simple, scalable proteomic imaging for high-dimensional profiling of intact systems.* Cell, 2015. **163**(6): p. 1500-14.
20. Staudt, T., et al., *2,2'-thiodiethanol: a new water soluble mounting medium for high resolution optical microscopy.* Microscopy Research & Technique, 2007. **70**(1): p. 1-9.
21. Chozinski, T.J., et al., *Expansion microscopy with conventional antibodies and fluorescent proteins.* Nature Methods, 2016. **13**(6): p. 485-8.
22. Chen, F., P.W. Tillberg, and E.S. Boyden, *Optical imaging. Expansion microscopy.* Science, 2015. **347**(6221): p. 543-8.
23. Ku, T., et al., *Multiplexed and scalable super-resolution imaging of three-dimensional protein localization in size-adjustable tissues.* Nature Biotechnology, 2016. **34**(9): p. 973.
24. Tainaka, K., et al., *Chemical Landscape for Tissue Clearing Based on Hydrophilic Reagents.* Cell Reports, 2018. **24**(8): p. 2196-2210.e9.
25. Dean, K., et al., *Deconvolution-free Subcellular Imaging with Axially Swept Light Sheet Microscopy*, in *Biophys J*. 2015. p. 2807-15.

26. Kumar, A., et al., *Dual-view plane illumination microscopy for rapid and spatially isotropic imaging.* Nature Protocols, 2014. **9**: p. 2555-2573.
27. Migliori, B., et al., *Light sheet theta microscopy for rapid high-resolution imaging of large biological samples.* BMC Biology, 2018. **16**(1): p. 57.
28. Glaser, A.K., et al., *Light-sheet microscopy for slide-free non-destructive pathology of large clinical specimens.* Nature Biomedical Engineering, 2017. **1**(7): p. 0084.
29. Hayashi, K., *Microscope Objective*, O.O.C. Ltd, Editor. 1999, Olympus Optical Co Ltd: Japan.
30. Glaser, A.K., et al., *Multidirectional digital scanned light-sheet microscopy enables uniform fluorescence excitation and contrast-enhanced imaging.* Scientific Reports, 2018. **8**(1): p. 13878.
31. Hörl, D., et al., *BigStitcher: Reconstructing high-resolution image datasets of cleared and expanded samples.* bioRxiv, 2018.
32. Balazs, B., et al., *A real-time compression library for microscopy images.* bioRxiv, 2017.
33. Pietzsch, T., et al., *BigDataViewer: visualization and processing for large image data sets.* Nature Methods, 2015. **12**(6): p. 481-3.
34. Schneider, C.A., W.S. Rasband, and K.W. Eliceiri, *NIH Image to ImageJ: 25 years of image analysis.* Nature Methods, 2012. **9**(7): p. 671.
35. Krauter, P., et al., *Optical phantoms with adjustable subdiffusive scattering parameters.* Journal of Biomedical Optics, 2015. **20**(10): p. 105008.
36. Malitson, I.H., *Interspecimen Comparison of the Refractive Index of Fused Silica*,†.* Journal of the Optical Society of America, 1965. **55**(10): p. 1205-1209.
