# Supplementary material for: Multi-immersion open-top light-sheet microscope for high-throughput imaging of cleared tissues
Source: Nat Commun. 2019 Jul 4;10:2781. doi: 10.1038/s41467-019-10534-0 (PMC6609674; doi:10.1038/s41467-019-10534-0)
Supplement: Supplementary file 2 — Description of Additional Supplementary Files [file 41467_2019_10534_MOESM2_ESM.pdf]

Description of Additional Supplementary Files

File name: Supplementary Movie 1

Description: Video demonstrating the simplicity of mounting specimens on the OTLS system, as well as real-time previewing of specimen(s), followed by automated imaging of the specimen(s).

File name: Supplementary Movie 2

Description: Time-lapse video demonstrating the operating principles of the OTLS system (image tiling as well as multiple specimens). Shown are 12 ECi-cleared human prostate biopsies, stained with TO-PRO3 (nuclear) and Eosin (cytoplasmic).

File name: Supplementary Movie 3

Description: Surface blend volume rendering of one of the ECi-cleared human prostate biopsies shown in **Fig. 3a**. Color channels are TO-PRO3 (cyan) and Eosin (purple).

File name: Supplementary Movie 4

Description: Z-stack of benign glands in a ECi-cleared human prostate biopsy. The TO-PRO3 and Eosin channels are pseudo-colored to mimic conventional H&E staining.

File name: Supplementary Movie 5

Description: Maximum intensity volume rendering of a TDE-cleared GFP-expressing mouse brain slice.

File name: Supplementary Movie 6

Description: Maximum intensity volume renderings of the bronchial tree (EpCAM, shown in yellow) in a Ce3D-cleared mouse lung

File name: Supplementary Movie 7

Description: Z-stack of a small sub-region of an entire ExM-cleared mouse kidney specimen. Color channels are DAPI (white), WGA-lectin (magenta), Coll IV (cyan), and Podxl (green).

File name: Supplementary Movie 8

Description: Surface blend volume rendering of a small sub-region of an entire ExM-cleared mouse kidney specimen

File name: Supplementary Movie 9

Description: Fly through of a blood vessel for the expanded mouse kidney shown in Supplementary Videos 8 and 9.
