# Supplementary material for: Multi-immersion open-top light-sheet microscope for high-throughput imaging of cleared tissues
Source: Nat Commun. 2019 Jul 4;10:2781. doi: 10.1038/s41467-019-10534-0 (PMC6609674; doi:10.1038/s41467-019-10534-0)
Supplement: Supplementary file 12 — Reporting Summary [file 41467_2019_10534_MOESM12_ESM.pdf]

nature research

Corresponding author(s): Adam Glaser

Last updated by author(s): Apr 25, 2019

Reporting Summary

Nature Research wishes to improve the reproducibility of the work that we publish. This form provides structure for consistency and transparency in reporting. For further information on Nature Research policies, see Authors & Referees and the Editorial Policy Checklist.

Statistics

For all statistical analyses, confirm that the following items are present in the figure legend, table legend, main text, or Methods section.

n/a	Confirmed	
☒	☐	The exact sample size (*n*) for each experimental group/condition, given as a discrete number and unit of measurement
☒	☐	A statement on whether measurements were taken from distinct samples or whether the same sample was measured repeatedly
☒	☐	The statistical test(s) used AND whether they are one- or two-sided<br>*Only common tests should be described solely by name; describe more complex techniques in the Methods section.*
☒	☐	A description of all covariates tested
☒	☐	A description of any assumptions or corrections, such as tests of normality and adjustment for multiple comparisons
☒	☐	A full description of the statistical parameters including central tendency (e.g. means) or other basic estimates (e.g. regression coefficient) AND variation (e.g. standard deviation) or associated estimates of uncertainty (e.g. confidence intervals)
☒	☐	For null hypothesis testing, the test statistic (e.g. *F*, *t*, *r*) with confidence intervals, effect sizes, degrees of freedom and *P* value noted<br>*Give P values as exact values whenever suitable.*
☒	☐	For Bayesian analysis, information on the choice of priors and Markov chain Monte Carlo settings
☒	☐	For hierarchical and complex designs, identification of the appropriate level for tests and full reporting of outcomes
☒	☐	Estimates of effect sizes (e.g. Cohen's *d*, Pearson's *r*), indicating how they were calculated

Our web collection on statistics for biologists contains articles on many of the points above.

Software and code

Policy information about availability of computer code

Data collection	Data was collected using a custom in-house LABVIEW code for controlling the microscope system. The LABVIEW code uses device drivers from vendors for controlling the camera, microscope stage, laser package, filter wheel, and scanning mirror. This LABVIEW code is highly specific to the custom microscope system, and therefore is not included in the submission. However, it is available upon request.
Data analysis	Data is processed using a combination of commercial, open-source, and custom written code. Raw data files are read into subsequent processing routines using the DCIMG SDK from Hamamatsu (https://dcam-api.com/dcam-sdk-login/). We have compiled a DLL using this SDK (usable only on Windows platform). Open-source tools used include HDF5 (https://www.hdfgroup.org/solutions/hdf5/), a B3D compression filter for HDF5 files (https://git.embl.de/balazs/B3D), Python2 and required packages, and BigStitcher for visualizing and stitching the tiled imaging data (https://imagej.net/BigStitcher). Supplementary code is included in this manuscript submission, and example data and code usage instructions are described in the supplementary material file and README.

For manuscripts utilizing custom algorithms or software that are central to the research but not yet described in published literature, software must be made available to editors/reviewers. We strongly encourage code deposition in a community repository (e.g. GitHub). See the Nature Research guidelines for submitting code & software for further information.

Data

Policy information about availability of data

All manuscripts must include a data availability statement. This statement should provide the following information, where applicable:

- Accession codes, unique identifiers, or web links for publicly available datasets
- A list of figures that have associated raw data
- A description of any restrictions on data availability

Example imaging data for testing the provided code is available on FigShare: https://figshare.com/articles/Supplementary_Data/7685597. Full imaging datasets are available upon request.

Field-specific reporting

Please select the one below that is the best fit for your research. If you are not sure, read the appropriate sections before making your selection.

☒ Life sciences ☐ Behavioural & social sciences ☐ Ecological, evolutionary & environmental sciences

For a reference copy of the document with all sections, see nature.com/documents/nr-reporting-summary-flat.pdf

Life sciences study design

All studies must disclose on these points even when the disclosure is negative.

Sample size	N/A
Data exclusions	N/A
Replication	N/A
Randomization	N/A
Blinding	N/A

Reporting for specific materials, systems and methods

We require information from authors about some types of materials, experimental systems and methods used in many studies. Here, indicate whether each material, system or method listed is relevant to your study. If you are not sure if a list item applies to your research, read the appropriate section before selecting a response.

Materials & experimental systems

n/a	Involved in the study	
☐	☒	Antibodies
☒	☐	Eukaryotic cell lines
☒	☐	Palaeontology
☐	☒	Animals and other organisms
☒	☐	Human research participants
☒	☐	Clinical data

Methods

n/a	Involved in the study	
☒	☐	ChIP-seq
☒	☐	Flow cytometry
☒	☐	MRI-based neuroimaging

Antibodies

Antibodies used	CD3-BV421 (Cat: 100228, BioLegend)<br>B220-e660 (Cat: 50-0452-82, ThermoFisher)<br>Epcam-APC (Cat: 17-5791-82, ThermoFisher)<br>CK8-18 (Cat:MS743S0, ThermoFisher)<br>Goat anti-podocalyxin (Cat: AF1556, R&D Sys. Inc.)<br>Rabbit anti-collagen IV (cat: ab6586, Abcam)
Validation	CD3, B220, and Epcam-APC have been validated previously in the original Ce3D clearing publication.<br>https://www.ncbi.nlm.nih.gov/pubmed/28808033<br>The CK8-18 antibodies have been validated in a recent study by Van Royen et al.<br>https://www.ncbi.nlm.nih.gov/pubmed/27353346<br>The podocalyxin and collagen IV antibodies were validated previously by Chozinski et al.<br>https://www.nature.com/articles/s41598-018-28694-2

Animals and other organisms

Policy information about studies involving animals; ARRIVE guidelines recommended for reporting animal research

Laboratory animals	Mouse brain tissue was harvested from a mouse of line Sst-IRES-Cre;Ai139(TIT2L-GFP-ICL-TPT). Genotyping confirmed expression of Cre and tdTomato for this individual. The mouse was sacrificed at age P96 by trans-cardial perfusion with 4% PFA.
Wild animals	N/A
Field-collected samples	N/A

Ethics oversight | University of Washington and Allen Institute for Brain Science Institutional Care and Use Committees

Note that full information on the approval of the study protocol must also be provided in the manuscript.
